# Numeric social-media posts engage people with climate science

**DOI:** 10.1093/pnasnexus/pgae250

**Published:** 2024-06-29

**Authors:** Ellen Peters, David M Markowitz, Ariel Nadratowski, Brittany Shoots-Reinhard

**Affiliations:** Center for Science Communication Research, School of Journalism and Communication, University of Oregon, Eugene, OR 97403, USA; Psychology Department, University of Oregon, Eugene, OR 97403, USA; Center for Science Communication Research, School of Journalism and Communication, University of Oregon, Eugene, OR 97403, USA; Department of Communication, Michigan State University, East Lansing, MI, USA; Center for Science Communication Research, School of Journalism and Communication, University of Oregon, Eugene, OR 97403, USA; Center for Science Communication Research, School of Journalism and Communication, University of Oregon, Eugene, OR 97403, USA; Department of Psychology, The Ohio State University, Columbus, OH 43210, USA

**Keywords:** social media, engagement, climate change, trust, decision making

## Abstract

Innumeracy (lack of math skills) among nonscientists often leads climate scientists and others to avoid communicating numbers due to concerns that the public will not understand them and may disengage. However, people often report preferring to receive numbers; providing them also can improve decisions. Here, we demonstrated that the presence vs. absence of at least one Arabic integer in climate-related social-media posts increased sharing up to 31.7% but, counter to hypothesis, decreased liking of messages 5.2% in two preregistered observational studies (climate scientists on Twitter, *N*  *>* 8 million Tweets; climate subreddit, *N*  *>* 17,000 posts and comments). We speculated that the decreased liking was due, not to reduced engagement, but to more negative feelings towards climate-related content described with numeric precision. A preregistered within-participant experiment (*N* = 212) then varied whether climate consequences were described using Arabic integers (e.g. “90%”) or another format (e.g. verbal terms, “almost all”). The presence of Arabic integers about consequences led to more sharing, wanting to find out more, and greater trust and perceptions of an expert messenger; perceived trust and expertise appeared to mediate effects on sharing and wanting to find out more. Arabic integers about consequences again led to more negative feelings about the Tweets as if numbers clarified the dismaying magnitude of climate threats. Our results indicate that harnessing the power of numbers could increase public trust and concern regarding this defining issue of our time. Communicators, however, should also consider counteracting associated negative feelings—that could halt action—by providing feasible solutions to increase people's self-efficacy.

**Significance Statement**

Climate science posted on social media may reach more citizens and scientists, but only if people engage. We investigated the impact of including numbers in climate-related posts vs. not including them. Results of two preregistered observational studies on social-media sites revealed that people shared social-media posts with numbers more but liked them less. A preregistered experiment then documented that posts describing climate consequences with numbers led to more sharing and also greater trust and perceptions of an expert messenger, but more negative feelings. Scientists describing climate consequences might want to harness the power of numbers to increase public concern but should consider counteracting associated negative feelings—that could halt action—by providing feasible solutions to increase people's feelings of efficacy.

## Introduction

A primary purpose of science is to quantify natural phenomena, including about climate change. Thus, understanding climate science often requires engaging with, understanding, and using numeric information. But therein lies a problem: many nonscientists are innumerate and may turn away from science described numerically. As a result, instead of saying that “Earth's temperature has risen by an average of 0.14° Fahrenheit per decade since 1880” (1), stating the less precise alternative, it “has risen steadily over time” might appeal more. Against this backdrop, in two observational studies on social-media and one experimental study—all in the context of climate change—we examined whether numbers engage or disengage people in information-rich environments.

American views on climate change are quickly changing. More than half of Americans (58%) now believe that global warming is happening and is mostly human-caused; 64% are at least “somewhat worried” about it (2). However, a similar proportion (54%) may not know the latest climate-science news (3). One possible opportunity is to meet people where they are, particularly online, and use social media as a venue for climate scientists and the public to interact with climate science. This idea has potential, given that 75% of US adults use social media (4) and 50% reported getting news, including science news, at least sometimes from it (5). Furthermore, social media makes information, including historical information, available to the masses and can allow people to connect with individuals who ordinarily are unreachable, such as climate scientists (6). Thus, science posted on social media, if people engage (7), may reach more citizens and scientists alike, potentially increasing science literacy and impact while making climate science more accessible, equitable, and actionable; it also may benefit researchers through increases in reputation and career progress (8).

Many social-media posts, however, see little to no engagement (9). More engagement emerges when scientific posts arouse emotion, make the work seem more useful or interesting, or reflect positively on the sender (10). Having more followers and including an image also increase engagement (11). Unstudied is the effect of providing information with Arabic numbers (written symbols for numerals, e.g. “3” but not “three” or “several”) on social-media despite the importance of numeric climate-change consequences.

On the one hand, the presence of Arabic numbers may decrease engagement. This prediction is based on an innumerate and math-anxious public (almost one-third of US adults are functionally innumerate; 12, 13) faced with abstract climate numbers (1.5 °C, 27% of greenhouse emissions) that are unclearly linked with their daily decisions. On the other hand, people often prefer receiving numeric information and find it useful (14, 15). In economics and public policy, having such information may help people pay attention and find or demand options that best suit their preferences (16, 17). Providing numbers also can help people make better choices. Their provision decreases overestimation of medical risks and increases willingness to take medications and vaccines compared to not providing them (18, 19). If people prefer getting numbers and recognize their benefits, social-media posts with numbers may be more engaging than those without numbers.

In this article, we focus on two preregistered, hypothesized effects of numbers on perceptions of messages that contain them. First, people expect experts to be more likely to provide numbers than nonexperts (20). We therefore reasoned that providing numbers would lead to greater perceptions that a post came from an expert. If true, this is important because we also know that posts perceived as from experts engage people disproportionately, even in politicized domains (21). Furthermore, people trust messages, such as from physicians that contain numbers more than the same messages without them (22, 23). Trusted messages and messengers then typically engage people more, including on climate issues (24). These trust and expertise data predict that people will engage more with numeric than nonnumeric climate posts.

## The current studies

The studies on perceived expertise and trust in numeric messages, however, come primarily from nonclimate contexts. Their results could be due to health professionals being trusted messengers (25). We know that providing climate-related numbers (vs. not providing them) improves understanding and interpretation of climate issues (26). Less clear is what might happen to engagement with climate issues on social-media, given declining trust in media and other institutions (27) and rising misinformation and polarization (28, 29). In three studies, we explored whether the positive effects of Arabic numbers might generalize to the question of engagement on social media with climate-change posts. We operationalized engagement through sharing, upvoting, or liking social-media posts. If numbers engage the public, this information might inform the social-media playbook for scientists and climate communicators who want to increase public concern and knowledge regarding this “defining issue of our time” (30).

Here, we performed observational studies on two social-media platforms (study 1a: Twitter [now X], study 1b: reddit) and conducted a controlled experiment. Using a randomly selected half of a curated *scientists-who-do-climate* Twitter list, we extracted all Tweet texts and engagements (i.e. likes, retweets) from each user (8,003,920 Tweets for preregistered analyses: https://aspredicted.org/blind.php?x=DF7_FZD); 23.48% contained at least one Arabic number and were considered numeric (*n*  *=* 1,879,182).^a^ We conducted another observational study using reddit to rule out platform effects and test generalizability, since Twitter is a microblogging site whereas reddit is a news aggregator and content rating site. We similarly extracted and processed all posts (*n*  *=* 962) and comments (*n*  *=* 16,539) from the r/climatechange subreddit from May to November, 2022 for preregistered analyses of whether people engaged more with climate content containing Arabic numbers.

**Fig. 1.**
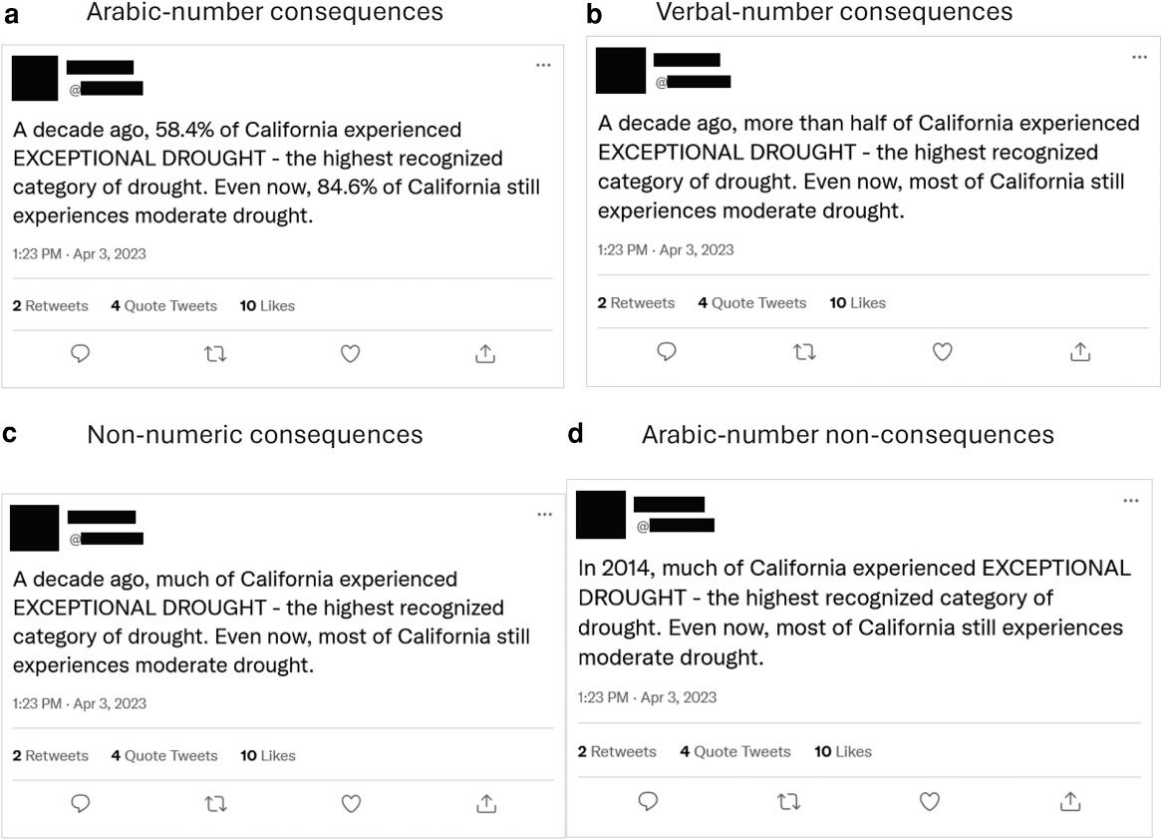
Tweet types: a) Arabic-number consequences, b) Verbal-number consequences, c) Nonnumeric consequences, and d) Arabic-number nonconsequences (which also contained nonnumeric consequences).

Although we analyzed social-media posts among climate experts (study 1a) and from a climate-dedicated forum (study 1b), we could not guarantee that all posts concerned climate nor that numbers referred to climate consequences (despite including exploratory covariates, e.g. each Tweet's proportion of climate-related terms). We also were unable to directly assess perceptions of posts with and without numbers. As a result, in study 2, we conducted a within-participant experiment of 20 Tweets to mimic the social-media milieu of messages; all Tweets focused on climate consequences (i.e. monetary costs or impacts on the earth, humans, or other species) and were tested in a general-public sample (preregistration: https://osf.io/md36r/). For each Tweet, participants (final *N* = 212) were randomly assigned to one of four Tweet types describing the same consequence(s). Numeric exactness of consequences decreased from one Tweet type to the next: Arabic-number consequences (e.g. 58.4%), verbal-number consequences (e.g. more than half), nonnumeric consequences (e.g. much of), or Arabic-number nonconsequences (i.e. ancillary information with at least one Arabic number, e.g. the year 2014) used to test the mere presence of Arabic numbers (Fig. 1 and Table S11). The latter two types had the same low consequence exactness. We considered manipulating numeric exactness of nonconsequences, too, but decided against it to reduce participant burden and because of the critical importance of communicating climate consequences.

We predicted that the presence of Arabic-number-consequence information would engage more than its absence because participants would trust the message more and perceive an expert messenger. We also explored whether other attitudes towards the message would be similarly positive and if Arabic-number-consequence Tweets would have larger effects among participants higher in objective numeracy, with greater number preferences, and with more liberal political views (who may agree more and react less to climate messages than conservatives) (22, 31, 32).

## Analytic plan

We controlled for factors related to people engaging more with social-media posts: message emotionality (proportion of words with positive emotion/tone and/or negative emotion/tone) and word count (10), a proxy for image presence, and number of followers (only in Twitter study 1a; reddit does not have follower counts) (11); we further controlled for the quantity of climate-related words and verbal numeric descriptors (e.g. *first*, *million*) in each message. Results with positivity (proportion of words with positive emotion)—instead of emotionality—are included in the latter half of model-results tables; Tweet positivity lacked variance in Study 2 and was not analyzed. Preregistered analyses (controlling for objective numeracy only) demonstrated similar results except on liking, which reversed direction (Tables S3, S8, and S16, Text S3). Throughout, all continuous predictors were standardized; b-coefficients are unstandardized.

## Results

### Study 1a—Twitter

Climate-scientist Tweets with Arabic numbers were shared more. They received 16.9% more retweets (*t* = 41.36, *P <* 0.001) and 10.5% more quote Tweets (*t* = 6.59, *P < 0*.001) than those without numbers. However, climate-scientist Tweets with vs. without numbers were liked 5.2% less often (*t* = −11.45, *P* < 0.001. See Table 1 for model results with controls and Tables S1 (full results) and S5 (intercorrelations).

**Table 1.** Studies 1a and 1b—unstandardized coefficients and their significance from the linear mixed models results for Twitter and Reddit engagement variables.

	Retweets—study 1a		Quote Tweetsstudy 1a		Likes—study 1a		Upvotes on posts^a^—study 1b		Upvotes on comments—study 1b	
	*b*(SE)	*P*	*b*(SE)	*P*	*b*(SE)	*P*	*b*(SE)	*P*	*b*(SE)	*P*
Intercept	0.146 (0.008)	*P* < 0.001	0.0003 (0.00003)	*P* < 0.001	0.00926 (0.0006)	*P* < 0.001	0.319 (0.019)	*P* < 0.001	0.0624 (0.004)	*P* < 0.001
Contains number	0.0181 (0.0004)	*P* < 0.001	0.00007 (0.00001)	*P* < 0.001	−0.0009 (0.00008)	*P* < 0.001	0.0831 (0.038)	0.028	0.00277 (0.002)	0.140
LIWC word count	0.0067 (0.0002)	*P* < 0.001	0.0005 (0.000005)	*P* < 0.001	0.0106 (0.00004)	*P* < 0.001	0.0068 (0.016)	0.661	0.00338 (0.0009)	*P* < 0.001
LIWC emotionality	−0.0162 (0.0002)	*P* < 0.001	0.00007 (0.000005)	*P* < 0.001	0.0017 (0.00003)	*P* < 0.001	0.0337 (0.014)	0.017	−0.0011 (0.0008)	0.157
Climate words	−0.0032 (0.0002)	*P* < 0.001	0.00004 (0.000005)	*P* < 0.001	−0.00001 (0.0000)	*P* = 0.865	−0.0089 (0.015)	0.544	0.00203 (0.0008)	0.009
Verbal numeric terms	0.0070 (0.0002)	*P* < 0.001	0.00001 (0.000005)	*P* = 0.039	0.0001 (0.00003)	*P* < 0.001	0.0196 (0.014)	0.151	0.00127 (0.0008)	0.095
Contains weblink	−0.0666 (0.0004)	*P* < 0.001	0.00080 (0.00001)	*P* < 0.001	0.0162 (0.00007)	*P* < 0.001	N/A		0.00623 (0.0026)	0.017
Follower count	0.0023 (0.0158)	0.884	0.00111 (0.00007)	*P* < 0.001	0.0194 (0.001)	*P* < 0.001	N/A		N/A	
*Rm, Rc*	0.076, 0.373		0.096, 0.116		0.242, 0.291		0.108, 0.702		0.038, 0.804	

Using the MuMIn package in R, the Nakagawa *R*^2^m is the marginal *R*^2^ (variance explained by the fixed effects, only), and Nakagawa *R*^2^c is the conditional *R*^2^ (variance explained by the fixed and random effects). We then use R as a measure of variation because variance is less appropriate for use as a measure of effect size or explanatory power because variance is a squared measure, out of the dimensionality of the original measurements (33, 34). See Tables S1 and S6 for more complete results.

^a^No weblinks were found in posts and that variable was dropped from that model as was followers since reddit does not have them.

### Study 1b—reddit

Posts with Arabic numbers received 31.7% more estimated upvotes than posts without them (*t =* 2.20, *P = 0*.028). Comments with Arabic numbers, however, did not earn significantly more upvotes than those without them (*t* = 1.48, *P = 0*.140). See Table 1 for model results with controls and SI Appendix, Tables S6 (full results) and S10 (intercorrelations).

These results suggest that people engaged more with social-media posts containing Arabic numbers than those without them, even after controlling for engagement-related factors, verbal indicators of numeric consequences (e.g. *billions* of dollars), and each post's climate-relatedness. Posts containing more verbal-only numeric indicators of consequences also saw greater engagement. Most engagements occurred soon after posting, which could have skewed these results that relied on per-day averages over long periods; however, similar results emerged when controlling instead for time since post (Tables S2, S4, S7, and S9). Overall, the effect sizes were small, but we believe reasonable given heavy competition for users' online attention in the natural world. Importantly, numeric categorizations were based on the presence vs. absence of Arabic integers. Thus, numeric posts might have focused on quantified consequences (e.g. costs are $260 billion) or a nonconsequence number (e.g. the year 2020), but we do not know the effects of numeric and nonnumeric posts for the identical content given that our data came from the wild. We also do not know whether posts specifically concerned climate (although we controlled for the quantity of climate-related words), nor whether engagements indicated interactions with the public or scientist–scientist interactions. In study 2, we addressed these concerns by experimentally varying the numeric exactness of Tweets describing climate consequences and testing with a general-public sample. Additionally and counter to hypothesis, people liked Arabic-number Tweets less; we reasoned that the “heart” that indicates liking might confound engagement with feelings towards message content. In study 2, we explicitly asked participants about their feelings towards the message.

### Study 2—within-participant Tweet experiment

Participants were more likely to share Tweets containing Arabic-number consequences than other Tweet types (*b*_VerbalC:ArabicC_ = −0.112, SE = 0.033, *P* = 0.001, *b*_NonnumC:ArabicC_ = −0.120, SE = 0.036, *P* = 0.001, *b*_ArabicNC:ArabicC_ = −0.085, SE = 0.033, *P* = 0.011); they also wanted to find out more about them. For example, 20.5% of participants were at least “somewhat likely” to share Arabic-number-consequence Tweets vs. participants who saw verbal-number consequences, nonnumeric consequences, or Arabic-number nonconsequences Tweets (respectively, 17.2, 17.0, and 17.7%); more numerate participants were less likely to share Tweets (*b* = −0.262, SE = 0.068, *P* < 0.001). Further, compared to other Tweet types, Arabic-number-consequence Tweets were perceived as more trustworthy (*b*_VerbalC:ArabicC_ = −0.211, SE = 0.043, *P* < 0.001, *b*_NonnumC:ArabicC_ = −0.165, SE = 0.048, *P* = 0.001, *b*_ArabicNC:ArabicC_ = −0.105, SE = 0.043, *P* = 0.106) and likely to be from an expert (*b*_VerbalC:ArabicC_ = −0.488, SE = 0.041, *P* < 0.001, *b*_NonnumC:ArabicC_ = −0.554, SE = 0.046, *P* < 0.001, *b*_ArabicNC:ArabicC_ = −0.372, SE = 0.042, *P* < 0.001). Greater perceptions of trustworthiness and expertise mediated the positive effect of numbers' presence on sharing and finding out more in exploratory analyses (Text S1). Arabic-number-consequence Tweets also were perceived as more accurate, clear, and interesting. No significant effects emerged for Tweet emotionality or word count except that Tweets with higher vs. lower word counts were perceived as more likely from an expert (*b* = 0.017, SE = 0.008, *P* = 0.031). See Tables S12–S14 (full model results) and S19 (intercorrelations).

Consistent with our reasoning, participants reported more negative feelings about Arabic-number-consequence Tweets than nonnumeric-consequence Tweets (*b* = 0.077, SE = 0.038, *P* = 0.044); feelings about Arabic-number-consequence Tweets did not differ from the other two Tweet types. More vs. less numerate people felt less positive (*b* = −0.141, SE = 0.066, *P* = 0.034). Perceived trustworthiness and expertise did not mediate the effect on feelings (Text S1).

#### Moderators

Drawing on prior work (31), we tested for moderation by interacting Tweet type separately with objective numeracy, number preferences, and ideology in the mixed-effects regression models (we replaced subjective numeracy with its subscale number preferences as preregistered for exploration). In each analysis, the simple effects of each Tweet type vs. the Arabic-number-consequences condition remained substantially similar to those presented in Table 1. To foreshadow the effects shown in Text S2 and Table S15, people lower in numeracy and number preferences (vs. higher) and those more conservative (vs. liberal) were affected less by Arabic-number-consequences Tweets. Substituting climate change risk perceptions and affect for ideology resulted in substantially similar results.

## General discussion

One of the biggest challenges with climate-change communication is getting people to care and engage with the issues (35). In this article, results from Twitter climate scientists and a climate subreddit demonstrated a previously unstudied benefit of leading with numeric evidence, namely that people shared and upvoted messages more when they included at least one Arabic integer. These findings suggest people perceived messages with Arabic integers as making more positive contributions. It was ambiguous though whether all posts concerned climate and all numbers described climate consequences. Thus, experimental study 2 carefully controlled both climate and numeric content. Its results confirmed our observational results, further clarifying that Tweets containing consequences quantified with Arabic numbers caused people to share and want to find out more than those containing verbal-number consequences, nonnumeric consequences, or nonconsequence Arabic numbers (e.g. a year). This latter result points to the possibility that Study 1's numeric results may be stronger if we compared numeric-consequence posts to other social-media posts, including those that contained numeric nonconsequences (e.g. the year 2020). Mediation results pointed towards study 2's greater engagement being consistent with participants trusting Arabic-number-consequence Tweets more and perceiving them as more likely from an expert. Participants also had other more positive attitudes to them; greater sharing intentions may have emerged instead because people perceived them as more interesting and likely to make them appear knowledgeable, both of which increase sharing (10) and amplify messages (36). Nonetheless, only 23% of study 1a's Tweets using preregistered data contained an Arabic number, perhaps due to expert beliefs that the data were low quality or people would not understand them (37).

Study 1a's Twitter “likes” did not support our initial engagement hypothesis. We reasoned that “likes,” more than retweets, may reflect feelings about climate-change consequences (38). Consistent with this possibility, negative compared to neutral posts from three news agencies increased “likes” less than retweets (39). Similarly, study 2 participants rated their feelings about numeric-consequence Tweets as more negative than other Tweet types but still wanted to share them and find out more; they also found these posts to be more clear, interesting, and accurate vs. other Tweets. It is unclear whether the negative-feelings results emerged due to seeing unwanted numbers or because providing them clarified the dismaying magnitude of climate threats. The former explanation seems less likely given that our number-preferences measure was unrelated to feelings about the posts. The latter explanation seems more likely given that the more numerate (who understand numbers better) expressed more negative feelings especially towards Arabic-number-consequence Tweets. Thus, although we initially grouped sharing and liking as engagement metrics, they appear to serve different social and psychological functions that are differentially linked to the presence of Arabic integers. Finally, we suspect the nonsignificant results for upvotes of reddit comments with numbers may be because responses to comments contain less focused content than the original posts, with people going off-topic and even trolling other users (40), though we note the reddit results were more mixed across modeling approaches than Twitter results.

If one goal in the fight against climate change is to engage people more with its science so they attend to issues, providing numeric-consequence data using Arabic numbers in social-media posts may be beneficial. Because such messages are also trusted more, and trusted messages are typically more persuasive (41), people may follow message recommendations more, too. A potential problem is that these results could motivate social-media users to disseminate misinformation using numeric data; these and other dynamics should be explored. For example, research could vary Tweet truthfulness and presence/absence of numeric data, exploring whether patently false numeric Tweets also would be shared more (e.g. Figure 1’s Tweets could be used as is vs. substituting “Exceptional snow” for “Exceptional drought”). Examining the effects of verbal vs. numeric uncertainty on engagement would also be of interest (14, 18, 26).

That numeric-consequence Tweets were shared more but elicited more negative feelings also introduces the question: Are retweets or likes on Twitter/X more valuable? In terms of increased social-media exposure, the answer depends on current Twitter/X algorithms. To influence users who are exposed, however, the results point towards numeric retweets being more valuable based on their links with perceived trust and expertise. Communicators though should also consider accommodating the associated negative feelings, which may slow action to reduce climate risks; identifying actions that are doable and effective may counteract these feelings (42, 43).

Climate scientists also should consider individual differences when designing messages to engage. Those lower in numeracy, lower in number preferences, and conservatives—who stereotypically deny climate change—generally were less affected by Tweet type; those higher in numeracy and number preferences and liberals responded most positively to the Arabic-number-consequences Tweets. Arabic-number-consequences Tweets; however, generally did not harm people's attitudes towards or propensity to share messages, and, across individual differences, they were perceived as more likely from an expert.

Understanding the psychology of numbers may further improve climate-scientist impact (44) whether through the use of precise numbers (e.g. “2.8” vs. “2”; 45, 46), affirming people's values prior to presenting information (44), or presenting valued information using Arabic numbers (e.g. climate cost savings for conservatives). Finally, the effect of numbers may be due to their contrast against the remaining text—a pop-out effect that focuses attention on the numbers. If true, using written-out number terms (e.g. “three”) would have less effect than using Arabic integers (e.g. “3”) and “100% more” would work better than “doubled.” Study 2 results—comparing Arabic-number-consequence and verbal-number-consequence Tweets—support this conjecture, which deserves future research.

The present results do not inform about effects of using Arabic integers to describe nonconsequences. They also are noninformative about whether people reflected on provided numbers; instead, the numbers might have been used superficially as intuitive cues indicating trustworthiness. Thus, while we hope that providing numeric information will inform, correct misperceptions, and provide a more complete perspective, it is unclear if these effects emerged on social media. Open-ended responses in an earlier prescription-drug study; however, suggested that participants can reflect on provided statistics and correct misperceptions (18).

In the wild, we also think that social-media users would share posts containing Arabic-number consequences more than other posts, but the present studies are not definitive. We also do not know whether providing numeric evidence would promote greater action on climate as it did in earlier studies on prescription-drug and vaccine uptake.

Those who question whether the public can handle numbers are correct in two ways—people liked them less and may not have always understood them. However, communicators may overlook that numeric information can engage people and elicit perceptions of trustworthiness and expertise critical to adopting behaviors. Although greater knowledge is one aim of science communication, it should also aim at creating long-term trust in science and scientists as a public good. Then, thinking more strategically about how numeric data are presented—including with feasible actions for people to take—would produce greater gains in people's understanding of benefits, risks, and other costs that presumably would allow them to make more sound choices consistent with the data and their own values (44, 47).

### Data sharing statement

Deidentified datasets and code for reproducibility purposes are at https://osf.io/md36r/.

## Methods

### Study 1a—Twitter field study

To obtain climate-scientist Tweets, we used a curated Twitter list *scientists-who-do-climate*, with over 3,000 climate scientists. We randomly selected half the scientists (mean [median] followers ∼ 3,200 [928]) and extracted their full Twitter archive including each Tweet's text and engagements (e.g. likes, retweets) through 2022 September 17 using the academic Twitter API (48). We obtained 8,003,920 Tweets from 1,598 unique climate-scientist Twitter accounts for preregistered analyses. We also gathered each Tweeter's number of followers and the presence/absence of any link in the Tweet (present = 2,756,447; 34.44% using the full data) as a proxy for an image/visual.

To identify Tweets with numbers, we excluded irrelevant text strings that may also contain numeric information. Thus, we excluded Twitter handles and URLs that might contain numeric information (@arvindpawan1) to the best of our ability. HTML tags and accented characters that may be converted to numbers also were removed to the best of our ability. After data cleaning, we then classified texts as numeric or nonnumeric. A minority of Tweets were numeric (23.48%, *n*  *=* 1,879,182).

To demonstrate the cleaning process, consider the following: An original, unprocessed Tweet stated “*Over 1 million km2 mapped with sonar by @NOAA's Okeanos!*  https://t.co/9l63BTaQeE.” Our automated cleaning procedures resulted in “Over 1 million km2 mapped with sonar by ’s Okeanos!” Here, the Twitter handle and URL were removed. This Tweet would be counted as numeric because it contained the numerals “1” (1 million) and “2” (km^2^).

Automated text analysis also was conducted for exploratory analyses using the well-established LIWC computer program (49) to count each Tweet's number of words and quantify its emotionality (positive emotion and tone [e.g. love, nice] plus negative emotion and tone [e.g. hurt, ugly]), and positivity (only positive emotion). We further created two dictionaries to calculate each message's rate of climate-related words (based on terms curated by ClimateWords.org; *n*  *=* 542 words) and verbal descriptions of numbers (e.g. *one* and *million*; *n* = 42 words from LIWC's “numbers” category, excluding those with Arabic integers; see https://osf.io/md36r/), controlling for both as a percentage of the total word count. However, we excluded Tweets containing any words from the climate-related word list that included an Arabic integer (e.g. 2050, 1.5 °C). Thus, the sample reduced slightly (∼7.8 million Tweets). LIWC analyses occurred on the processed texts (e.g. those with Twitter handles and URLs removed, etc.).

#### Analytic plan

Consistent with prior work (50), we transformed our dependent engagement variable to account for time because older Tweets might naturally have more engagements than newer ones. We subtracted the posted date from the final extraction date (2022 September 17) to create a difference score (the number of days between posting and extraction). Then, we individually divided likes, retweets, and quote Tweets by this date difference to create separate likes-per-day, retweets-per-day, and quote-Tweets-per-day metrics, and natural-log transformed each value using the formula *ln*(X + 1) out of skewness concerns.

We used linear mixed models (51, 52)—controlling for Tweet author as a random intercept due to nonindependence—to evaluate the relation between our binary independent variable (1 = numeric, 0 = nonnumeric) and likes, retweets, and quote Tweets. In each exploratory (not preregistered) analysis, we also controlled for LIWC word count, LIWC emotionality (in the main text; LIWC positivity in Supplementary Appendix), weblink presence, percentages of climate-related words and verbal descriptions of numbers, and each Twitter user's number of followers.

Inclusion of these reviewer-recommended covariates was based on prior work demonstrating verbal characteristics associated with online content virality (10), and visuals being more engaging than text alone (53). Although nearly impossible to control for all possible covariates when using naturally occurring data (54), we included covariates that were theoretically justifiable and available to us. Continuous variables were standardized in all studies. To calculate the percentage greater engagement with numeric than nonnumeric posts, we took the log-transformed estimated marginal means from the formula *ln*(X + 1), exponentiated the result, and subtracted one to create untransformed estimated marginal means (*e*^*M* − 1) where *M* = the target marginal mean. Then, we used the formula (*M*_Num_ − *M*_NonNum_)/(*M*_NonNum_) to create the percentage difference score. We present all preregistered analyses in Table S3. In Tables S2, S4, S7, and S9, we offer an alternative modeling approach for observational studies 1a and 1b, in which we natural log-transformed the dependent variable in each model and controlled for the date difference as a fixed effect. The results were substantively unchanged.

#### Study 1b—reddit field study

Using the RedditExtractoR package (55), all posts (*n*  *=* 962) and comments (*n*  *=* 16,539) were extracted from the 3,678 unique reddit authors in the *r*/climate change subreddit (May 2022 to November 2022). We used study 1a's preprocessing procedures. Out of the 17,501 total reddit texts for the preregistered analyses, 31.92% were numeric (*n* = 5,588).

We again subtracted the date of the post/comment from the date of data extraction (November 29, 2022) and created a difference score. We then divided the number of upvotes by this date difference to create an upvotes-per-day engagement metric. Finally, we natural-log-transformed this value using the formula *ln*(X + 1).

Our analytic plan followed study 1a; we related the independent variable (1 = numeric, 0 = nonnumeric) with upvotes and controlled for author as a random intercept in separate models for posts and comments. In the comments model, we also controlled for the comment thread with a random intercept due to their nonindependence and excluded posts and comments with negative upvotes. In each analysis, we controlled for LIWC word count, LIWC emotionality (weblink presence had variance only for comments; no follower count data existed on reddit), and each message's percentages of climate-related words and verbal number descriptions. To calculate the percentage greater engagement for numeric over nonnumeric posts, we exponentiated the predicted values for numeric and nonnumeric engagements, subtracted one, and calculated the percentage difference (like in study 1a).

### Study 2—Tweet experiment

#### Procedure

We preregistered a completely within-participants experiment, https://osf.io/md36r/. For it, we recruited participants (*N* = 250; *n* = 212 [85%] were retained) from a baseline cohort conducted 1–2 weeks earlier on Amazon's Mechanical Turk through CloudResearch based on a priori power analysis (*f*  *=* 0.10, *α* = 0.05, 80% power). About 50 participants were required for the main effect of Tweet type; we then oversampled to ensure enough participants to test interaction effects and covariates. CloudResearch was chosen due to high data quality in our past studies and relative to other data vendors (56). To ensure high quality participants, we recruited participants to the baseline cohort by requiring a 99–100% HIT approval rating and completion of at least 1,000 HITS, age over 18, and being within the United States. In the baseline survey, we collected information including about potential moderators, e.g. participants' objective numeracy, number preferences, climate-change affect and risk perceptions, and political ideology.

Participants were shown 20 Tweets in random order and asked questions about their feelings about the Tweet, likelihood to share it, and likelihood to want to find out more about the topic. They were also asked how trustworthy, clear, interesting, and accurate each Tweet was. At session's end, they completed a scientific reasoning measure (57). We used LIWC to determine word count, emotionality, and positivity of each Tweet.

### Materials

We wrote 20 Tweets and asked participants to respond to a random order of them. Tweets were written based on accurate numeric information about climate change and constructed to look like Tweets using https://www.tweetgen.com/create/tweet.html; identifying information about the hypothetical Tweeter was redacted (see Fig. 1 and Table S11). The average numbers of retweets, quote Tweets, and likes for each Tweet was set at 3, 4, and 10, respectively, with a random jitter of up to ±2.

Tweet format was manipulated (Arabic-number consequences, Verbal-number consequences, Nonnumeric consequences, and Arabic-number nonconsequences) and randomly assigned for each Tweet by participant so that the average participant saw about five Tweets from each Tweet format.

### Measures

For each Tweet, participants responded to seven questions: Feelings as a proxy for Twitter likes (“How do you feel about the tweet?” 1 = extremely negative, 2 = somewhat negative, 3 = slightly negative, 4 = slightly positive, 5 = somewhat positive, 6 = extremely positive) and likelihoods to share and find out more (“If you came across this tweet, how likely would you share it with others?” and “If you came across this tweet, how likely would you want to find out more about the topic?”; 1 = Extremely unlikely, 2 = Somewhat unlikely, 3 = Neither likely nor unlikely, 4 = Somewhat likely, 5 = Extremely likely). Then, using a matrix format, we asked “What are your opinions about the tweet you just saw? It is ____.” They responded to four word pairs presented in random order “untrustworthy–trustworthy”, “confusing–clear”, “boring–interesting”, and “biased–unbiased” on six-point scales, with higher numbers indicating more positive responses. Lastly, they responded to “How likely do you think it is that this tweet came from a nonexpert vs. an expert?” on a six-point scale (1 = Extremely likely from a nonexpert to 6 = Extremely likely from an expert).

In the baseline session, we assessed individual differences such as objective numeracy, number preferences, and political ideology. See Text S2 for measures and analysis results.

### Data cleaning and analytic plan

All participants were included in analyses. Using Rstudio, we fit mixed-effects regressions of each engagement variable (likelihood to share, likelihood to find out more, feelings about the Tweet), the Tweet's perceived trustworthiness, and likelihood that it came from an expert. We further explored other opinion word pairs (biased/accurate, confusing/clear, how feel, boring/interesting). We allowed for fixed effects of Tweet type and objective numeracy and random intercepts for individual participants and Tweets. In each analysis, we also controlled for LIWC word count and LIWC emotionality (LIWC positivity lacked variance). Using similar mixed-effects regressions, we further explored three two-way interactions of Tweet type with objective numeracy, number preferences, and ideology as fixed effects.

Studies were approved by the University of Oregon's Institutional Review Board (11182019.027). Informed consent was obtained from study 2 participants.

## Supplementary Material

pgae250_Supplementary_Data

## Note

a Exploratory analyses with additional covariates are based on a smaller sample size due to missing data (*N=*7,844,994). See Method.

## Preprint

This manuscript was posted as a preprint: https://www.researchsquare.com/article/rs-2944718/v1.
