# Supplementary material for: Numeric social-media posts engage people with climate science
Source: PNAS Nexus. 2024 Jun 29;3(7):pgae250. doi: 10.1093/pnasnexus/pgae250 (PMC11263877; doi:10.1093/pnasnexus/pgae250)
Supplement: pgae250_Supplementary_Data [file pgae250_supplementary_data.docx]

Supporting information

Numeric social-media posts engage people with climate science

Ellen Peters, PhD, David M. Markowitz, PhD*, Ariel Nadratowski, BS, & Brittany Shoots-Reinhard, PhD

*correspondence to: dmm@msu.edu

**This PDF file includes:**

Table of Contents

Supplementary Text

Supplementary Figures

Supplementary Tables

References for SI reference citations

Table of Contents

[Table S1. Full Covariate Model Results for Study 1a: Twitter Data 4](#_Toc164841421)

[Table S3. Full Preregistered Model Results for Study 1a: Twitter Data 12](#_Toc164841422)

[Table S4. Full Preregistered Model with Reconstructed DV for Study 1a: Twitter Data 13](#_Toc164841423)

[Table S5. Intercorrelations Among Variables for Study 1a: Twitter 14](#_Toc164841424)

[Table S6. Full Covariate Model Results for Study 1b: reddit Data 15](#_Toc164841425)

[Table S7. Full Covariate Model with Reconstructed DV for Study 1b: reddit Data 17](#_Toc164841426)

[Table S8. Full Preregistered Model Results for Study 1b: reddit Data 19](#_Toc164841427)

[Table S9. Full Preregistered Model with Reconstructed DV for Study 1b: reddit Data 20](#_Toc164841428)

[Table S10. Intercorrelations Among Variables for Study 1b: reddit 21](#_Toc164841429)

[Table S11. Stimuli in Study 2: Within-Participants Experiment 23](#_Toc164841430)

[Table S12. Full Covariate Model Results for Study 2: Within-Participants Experiment 27](#_Toc164841431)

[Table S13. Estimated marginal means by Condition for Study 2 28](#_Toc164841432)

[Table S14. Full Results for Condition Comparisons for Study 2 29](#_Toc164841433)

[Supplemental Text 1. Exploratory Mediation Analyses for Study 2 33](#_Toc164841434)

[Supplemental Text 2. Moderator analyses for Study 2: Within-Participant Tweet Experiment 35](#_Toc164841435)

[Table S15. Full Moderator Model Results for Study 2: Within-Participants Experiment 37](#_Toc164841436)

[Fig. S1. Moderation Analyses of Perceptions of Tweet Coming from an Expert by Objective Numeracy, Number Preferences, and Ideology for Study 2. 39](#_Toc164841437)

[Fig. S2. Moderation Analysis of Sharing by Ideology for Study 2. 40](#_Toc164841438)

[Supplemental Text 3. Preregistered Analyses for Study 2: Within-Participant Tweet Experiment 41](#_Toc164841440)

[Table S16. Full Preregistered Model Results for Study 2 42](#_Toc164841441)

[Table S17. Estimated marginal means (SE) in preregistered analyses for Study 2 43](#_Toc164841442)

[Table S18. Full Estimated Means from Preregistered Model Results for Study 2. 44](#_Toc164841443)

[Supplemental Text 4. Preregistered Analyses of Moderators of Tweet-type Effects for Study 2 48](#_Toc164841444)

[Fig. S3. Preregistered Moderation Analyses for Study 2. 49](#_Toc164841445)

[Fig. S4. Preregistered Additional Moderation Analyses for Study 2. 50](#_Toc164841446)

[Table S19. Intercorrelations Among All Study 2 Variables 51](#_Toc164841447)

[References 52](#_Toc164841448)

# **Table S1. Full Covariate Model Results for Study 1a: Twitter Data**

| DV: Log-transformed Retweets | | | | | | | |  |  |
| --- | --- | --- | --- | --- | --- | --- | --- | --- | --- |
| Fixed effects | *B* | *SE* | *df* | | *t* | | *p* | | |
| (Intercept) | 1.46E-01 | 7.50E-03 | 1.43E+03 | | 19.52 | | < .001 | | |
| Numeric tweets | 1.81E-02 | 4.38E-04 | 7.84E+06 | | 41.36 | | < .001 | | |
| LIWC Word count | 6.73E-03 | 1.97E-04 | 7.85E+06 | | 34.14 | | < .001 | | |
| LIWC Emotionality | -1.62E-02 | 1.85E-04 | 7.84E+06 | | -87.74 | | < .001 | | |
| Climate words | -3.23E-03 | 1.82E-04 | 7.84E+06 | | -17.71 | | < .001 | | |
| LIWC Verbal numeric terms | 6.97E-03 | 1.78E-04 | 7.84E+06 | | 39.16 | | < .001 | | |
| Contains weblink (URL link: Yes) | -6.66E-02 | 3.90E-04 | 7.85E+06 | | -171.07 | | < .001 | | |
| Follower count | 2.30E-03 | 1.58E-02 | 1.42E+03 | | 0.15 | | .884 | | |
| Random intercepts | *n* | σ^2^ | *SD* | |  | |  | | |
| Username | 1598 | .038 | .196 | |  | |  | | |
| Total number of observations, *Rm, Rc* | 7844994 | *Rm* = .076 | *R*c = .373 | |  | |  | | |
|  |  |  |  | |  | |  | | |
| DV: Log-transformed Quote Tweets | | | | | | | |  |  |
| Fixed effects | *B* | *SE* | *df* | *t* | | *p* | | |  |
| (Intercept) | 3.18E-04 | 3.48E-05 | 9.70E+02 | 9.16 | | < .001 | | |  |
| Numeric tweets | 7.49E-05 | 1.14E-05 | 7.80E+06 | 6.59 | | < .001 | | |  |
| LIWC Word count | 5.07E-04 | 5.11E-06 | 7.30E+06 | 99.17 | | < .001 | | |  |
| LIWC Emotionality | 6.53E-05 | 4.81E-06 | 7.80E+06 | 13.59 | | < .001 | | |  |
| Climate words | 3.76E-05 | 4.73E-06 | 7.72E+06 | 7.94 | | < .001 | | |  |
| LIWC Verbal numeric terms | 9.54E-06 | 4.63E-06 | 7.85E+06 | 2.06 | | .039 | | |  |
| Contains weblink (URL link: Yes) | 8.00E-04 | 1.01E-05 | 7.35E+06 | 79.14 | | < .001 | | |  |
| Follower count | 1.11E-03 | 7.09E-05 | 8.62E+02 | 15.59 | | < .001 | | |  |
| Random intercepts | *n* | σ^2^ | *SD* |  | |  | | |  |
| Username | 1598 | .000 | .001 |  | |  | | |  |
| Total number of observations, *Rm, Rc* | 7844994 | *Rm* = .096 | *R*c = .116 |  | |  | | |  |
|  |  |  |  |  | |  | | |  |
| DV: Log-transformed Likes | | | | | | | |  |  |
| Fixed effects | *B* | *SE* | *df* | *t* | | *p* | | |  |
| (Intercept) | 9.26E-03 | 5.80E-04 | 1.29E+03 | 15.96 | | < .001 | | |  |
| Numeric tweets | -9.00E-04 | 7.86E-05 | 7.85E+06 | -11.45 | | < .001 | | |  |
| LIWC Word count | 1.06E-02 | 3.54E-05 | 7.84E+06 | 298.93 | | < .001 | | |  |
| LIWC Emotionality | 1.66E-03 | 3.32E-05 | 7.85E+06 | 49.94 | | < .001 | | |  |
| Climate words | -5.58E-06 | 3.27E-05 | 7.84E+06 | -0.17 | | .865 | | |  |
| LIWC Verbal numeric terms | 1.23E-04 | 3.20E-05 | 7.84E+06 | 3.84 | | < .001 | | |  |
| Contains weblink (URL link: Yes) | 1.62E-02 | 7.00E-05 | 7.84E+06 | 231.08 | | < .001 | | |  |
| Follower count | 1.94E-02 | 1.22E-03 | 1.25E+03 | 15.98 | | < .001 | | |  |
| Random intercepts | *n* | σ^2^ | *SD* |  | |  | | |  |
| Username | 1598 | .000 | .015 |  | |  | | |  |
| Total # of observations, *Rm, Rc* | 7844994 | *Rm* = .242 | *R*c = .291 |  | |  | | |  |
|  |  |  |  |  | |  | | |  |
|  |  |  |  |  | |  | | |  |
| DV: Log-transformed Retweets | | | | | | | |  |  |
| Fixed effects | *B* | *SE* | *df* | *t* | | *p* | | |  |
| (Intercept) | 1.46E-01 | 7.50E-03 | 1.43E+03 | 19.45 | | < .001 | | |  |
| Numeric tweets | 1.97E-02 | 4.37E-04 | 7.84E+06 | 45.08 | | < .001 | | |  |
| LIWC Word count | 8.13E-03 | 1.95E-04 | 7.85E+06 | 41.71 | | < .001 | | |  |
| LIWC Positivity | -1.38E-02 | 1.81E-04 | 7.84E+06 | -75.80 | | < .001 | | |  |
| Climate words | -3.12E-03 | 1.82E-04 | 7.84E+06 | -17.10 | | < .001 | | |  |
| LIWC Verbal numeric terms | 7.35E-03 | 1.78E-04 | 7.84E+06 | 41.31 | | < .001 | | |  |
| Contains weblink (URL link: Yes) | -6.60E-02 | 3.89E-04 | 7.85E+06 | -169.48 | | < .001 | | |  |
| Follower count | 1.96E-03 | 1.58E-02 | 1.42E+03 | 0.12 | | .902 | | |  |
| Random intercepts | *n* | σ^2^ | *SD* |  | |  | | |  |
| Username | 1598 | .038 | .196 |  | |  | | |  |
| Total # of observations, *Rm, Rc* | 7844994 | *Rm* = .074 | *R*c = .373 |  | |  | | |  |
|  |  |  |  |  | |  | | |  |
| DV: Log-transformed Quote Tweets | | | | | | | |  |  |
| Fixed effects | *B* | *SE* | *df* | *t* | | *p* | | |  |
| (Intercept) | 3.22E-04 | 3.48E-05 | 9.70E+02 | 9.26 | | < .001 | | |  |
| Numeric tweets | 6.76E-05 | 1.13E-05 | 7.79E+06 | 5.96 | | < .001 | | |  |
| LIWC Word count | 5.00E-04 | 5.06E-06 | 7.32E+06 | 98.80 | | < .001 | | |  |
| LIWC Positivity | 4.50E-05 | 4.71E-06 | 7.82E+06 | 9.56 | | < .001 | | |  |
| Climate words | 3.68E-05 | 4.73E-06 | 7.72E+06 | 7.78 | | < .001 | | |  |
| LIWC Verbal numeric terms | 7.88E-06 | 4.63E-06 | 7.85E+06 | 1.70 | | .088 | | |  |
| Contains weblink (URL link: Yes) | 7.96E-04 | 1.01E-05 | 7.35E+06 | 78.82 | | < .001 | | |  |
| Follower count | 1.11E-03 | 7.09E-05 | 8.62E+02 | 15.60 | | < .001 | | |  |
| Random intercepts | *n* | σ^2^ | *SD* |  | |  | | |  |
| Username | 1598 | .000 | .001 |  | |  | | |  |
| Total # of observations, *Rm, Rc* | 7844994 | *Rm* = .095 | *R*c = .116 |  | |  | | |  |
| \| DV: Log-transformed Likes \| \| \| \| \| \| \| --- \| --- \| --- \| --- \| --- \| --- \| \| Fixed effects \| *B* \| *SE* \| *df* \| *t* \| *p* \| \| (Intercept) \| 9.34E-03 \| 5.81E-04 \| 1.29E+03 \| 16.08 \| < .001 \| \| Numeric tweets \| -1.08E-03 \| 7.85E-05 \| 7.85E+06 \| -13.74 \| < .001 \| \| LIWC Word count \| 1.04E-02 \| 3.50E-05 \| 7.84E+06 \| 297.16 \| < .001 \| \| LIWC Positivity \| 1.21E-03 \| 3.26E-05 \| 7.85E+06 \| 37.28 \| < .001 \| \| Climate words \| -2.28E-05 \| 3.27E-05 \| 7.84E+06 \| -0.70 \| .486 \| \| LIWC Verbal numeric terms \| 8.17E-05 \| 3.20E-05 \| 7.84E+06 \| 2.55 \| .011 \| \| Contains weblink (URL link: Yes) \| 1.61E-02 \| 6.99E-05 \| 7.84E+06 \| 229.96 \| < .001 \| \| Follower count \| 1.95E-02 \| 1.22E-03 \| 1.25E+03 \| 15.98 \| < .001 \| \| Random intercepts \| *n* \| σ^2^ \| *SD* \|  \|  \| \| Username \| 1598 \| .000 \| .015 \|  \|  \| \| Total # of observations, *Rm, Rc* \| 7844994 \| *Rm* = .242 \| *R*c = .290 \|  \|  \| \|  \|  \|  \|  \|  \|  \| | | | | | | | |  |  |

*Note*. All continuous variables were standardized (z-scored). The reference group for the binary “Numeric tweets” variable is “non-numeric tweets.” The reference group for the binary “URL link: Yes” variable is “URL link: No.”

**Table S2. Full Covariate Model with Reconstructed DV for Study 1a: Twitter Data**

| DV: Log-transformed Retweets | | | | | |
| --- | --- | --- | --- | --- | --- |
| Fixed effects | *B* | *SE* | *df* | *t* | *p* |
| (Intercept) | 1.55E+00 | 2.84E-02 | 1.55E+03 | 54.72 | <2e-16 |
| Numeric Tweets | 3.35E-01 | 1.76E-03 | 7.84E+06 | 190.35 | <2e-16 |
| LIWC word count | 7.71E-02 | 8.05E-04 | 7.85E+06 | 95.76 | <2e-16 |
| LIWC Emotionality | -1.83E-01 | 7.45E-04 | 7.84E+06 | -245.26 | <2e-16 |
| Climate words | 5.97E-02 | 7.34E-04 | 7.84E+06 | 81.36 | <2e-16 |
| LIWC Verbal numeric terms | 3.31E-02 | 7.17E-04 | 7.84E+06 | 46.19 | <2e-16 |
| Contains weblink (URL link: Yes) | -1.55E-01 | 1.58E-03 | 7.85E+06 | -98.34 | <2e-16 |
| Follower count | 5.50E-02 | 5.99E-02 | 1.53E+03 | 0.92 | .359 |
| Date difference | -2.33E-01 | 8.96E-04 | 7.83E+06 | -259.77 | <2e-16 |
| Random intercepts | *n* | σ^2^ | *SD* |  |  |
| Username | 1598 | .548 | .741 |  |  |
| Total # of observations, *Rm, Rc* | 7844994 | *Rm* = .182 | *Rc* = .387 |  |  |
|  |  |  |  |  |  |
| DV: Log-transformed Quote Tweets | | | | | |
| Fixed effects | *B* | *SE* | *df* | *t* | *p* |
| (Intercept) | 1.21E-02 | 1.17E-03 | 1.43E+03 | 10.31 | <2e-16 |
| Numeric Tweets | 8.61E-03 | 1.95E-04 | 7.84E+06 | 44.27 | <2e-16 |
| LIWC word count | 3.54E-02 | 8.88E-05 | 7.83E+06 | 398.23 | <2e-16 |
| LIWC Emotionality | 4.14E-03 | 8.22E-05 | 7.84E+06 | 50.30 | <2e-16 |
| Climate words | 2.34E-03 | 8.10E-05 | 7.84E+06 | 28.84 | <2e-16 |
| LIWC Verbal numeric terms | -3.37E-05 | 7.91E-05 | 7.84E+06 | -0.43 | .670 |
| Contains weblink (URL link: Yes) | 8.36E-02 | 1.74E-04 | 7.83E+06 | 480.87 | <2e-16 |
| Follower count | 5.52E-02 | 2.44E-03 | 1.38E+03 | 22.58 | <2e-16 |
| Date difference | -2.01E-02 | 9.87E-05 | 7.02E+06 | -203.30 | <2e-16 |
| Random intercepts | *n* | σ^2^ | *SD* |  |  |
| Username | 1598 | .001 | .030 |  |  |
| Total # of observations, *Rm, Rc* | 7844994 | *Rm* = .325 | *Rc* = .349 |  |  |
|  |  |  |  |  |  |
| DV: Log-transformed Likes | | | | | |
| Fixed effects | *B* | *SE* | *df* | *t* | *p* |
| (Intercept) | 3.56E-01 | 1.03E-02 | 1.53E+03 | 34.57 | <2e-16 |
| Numeric Tweets | -3.61E-02 | 7.26E-04 | 7.84E+06 | -49.79 | <2e-16 |
| LIWC word count | 2.32E-01 | 3.31E-04 | 7.85E+06 | 698.46 | <2e-16 |
| LIWC Emotionality | 5.79E-02 | 3.07E-04 | 7.84E+06 | 188.79 | <2e-16 |
| Climate words | -1.14E-02 | 3.02E-04 | 7.85E+06 | -37.85 | <2e-16 |
| LIWC Verbal numeric terms | 7.41E-04 | 2.95E-04 | 7.84E+06 | 2.51 | .012 |
| Contains weblink (URL link: Yes) | 6.03E-01 | 6.49E-04 | 7.85E+06 | 928.51 | <2e-16 |
| Follower count | 2.97E-01 | 2.17E-02 | 1.52E+03 | 13.67 | <2e-16 |
| Date difference | -2.43E-01 | 3.69E-04 | 7.82E+06 | -657.12 | <2e-16 |
| Random intercepts | *n* | σ^2^ | *SD* |  |  |
| Username | 1598 | .072 | .269 |  |  |
| Total # of observations, *Rm, Rc* | 7844994 | *Rm* = .506 | *Rc* = .572 |  |  |
|  |  |  |  |  |  |
| DV: Log-transformed Retweets | | | | | |
| Fixed effects | *B* | *SE* | *df* | *t* | *p* |
| (Intercept) | 1.55E+00 | 2.84E-02 | 1.55E+03 | 54.34 | <2e-16 |
| Numeric Tweets | 3.54E-01 | 1.76E-03 | 7.84E+06 | 200.90 | <2e-16 |
| LIWC word count | 9.45E-02 | 7.97E-04 | 7.85E+06 | 118.51 | <2e-16 |
| LIWC Positivity | -1.46E-01 | 7.31E-04 | 7.84E+06 | -200.31 | <2e-16 |
| Climate words | 6.11E-02 | 7.35E-04 | 7.84E+06 | 83.23 | <2e-16 |
| LIWC Verbal numeric terms | 3.75E-02 | 7.17E-04 | 7.84E+06 | 52.29 | <2e-16 |
| Contains weblink (URL link: Yes) | -1.47E-01 | 1.58E-03 | 7.85E+06 | -93.30 | <2e-16 |
| Follower count | 5.13E-02 | 6.00E-02 | 1.53E+03 | 0.86 | .393 |
| Date difference | -2.30E-01 | 8.97E-04 | 7.83E+06 | -256.57 | <2e-16 |
| Random intercepts | *n* | σ^2^ | *SD* |  |  |
| Username | 1598 | .551 | .742 |  |  |
| Total # of observations, *Rm, Rc* | 7844994 | *Rm* = .177 | *Rc* = .385 |  |  |
|  |  |  |  |  |  |
| DV: Log-transformed Quote Tweets | | | | | |
| Fixed effects | *B* | *SE* | *df* | *t* | *p* |
| (Intercept) | 1.23E-02 | 1.17E-03 | 1.43E+03 | 10.48 | <2e-16 |
| Numeric Tweets | 8.16E-03 | 1.94E-04 | 7.84E+06 | 42.02 | <2e-16 |
| LIWC word count | 3.49E-02 | 8.79E-05 | 7.83E+06 | 397.17 | <2e-16 |
| LIWC Positivity | 2.83E-03 | 8.06E-05 | 7.84E+06 | 35.09 | <2e-16 |
| Climate words | 2.29E-03 | 8.10E-05 | 7.84E+06 | 28.26 | <2e-16 |
| LIWC Verbal numeric terms | -1.40E-04 | 7.91E-05 | 7.84E+06 | -1.77 | .077 |
| Contains weblink (URL link: Yes) | 8.34E-02 | 1.74E-04 | 7.83E+06 | 479.81 | <2e-16 |
| Follower count | 5.52E-02 | 2.44E-03 | 1.38E+03 | 22.59 | <2e-16 |
| Date difference | -2.01E-02 | 9.87E-05 | 7.02E+06 | -204.10 | <2e-16 |
| Random intercepts | *n* | σ^2^ | *SD* |  |  |
| Username | 1598 | .001 | .030 |  |  |
| Total # of observations, *Rm, Rc* | 7844994 | *Rm* = .324 | *Rc* = .348 |  |  |
|  |  |  |  |  |  |
| DV: Log-transformed Likes | | | | | |
| Fixed effects | *B* | *SE* | *df* | *t* | *p* |
| (Intercept) | 3.59E-01 | 1.03E-02 | 1.53E+03 | 34.72 | <2e-16 |
| Numeric Tweets | -4.20E-02 | 7.25E-04 | 7.84E+06 | -57.93 | <2e-16 |
| LIWC word count | 2.26E-01 | 3.28E-04 | 7.85E+06 | 688.36 | <2e-16 |
| LIWC Positivity | 4.59E-02 | 3.01E-04 | 7.84E+06 | 152.64 | <2e-16 |
| Climate words | -1.19E-02 | 3.02E-04 | 7.85E+06 | -39.39 | <2e-16 |
| LIWC Verbal numeric terms | -6.61E-04 | 2.95E-04 | 7.84E+06 | -2.24 | .025 |
| Contains weblink (URL link: Yes) | 6.00E-01 | 6.49E-04 | 7.85E+06 | 924.45 | <2e-16 |
| Follower count | 2.98E-01 | 2.18E-02 | 1.52E+03 | 13.69 | <2e-16 |
| Date difference | -2.43E-01 | 3.69E-04 | 7.82E+06 | -659.05 | <2e-16 |
| Random intercepts | *n* | σ^2^ | *SD* |  |  |
| Username | 1598 | .073 | .270 |  |  |
| Total # of observations, *Rm, Rc* | 7844994 | *Rm* = .504 | *Rc* = .571 |  |  |
|  |  |  |  |  |  |

*Note*. All continuous variables were standardized (z-scored). The reference group for the binary “Numeric tweets” variable is “non-numeric tweets.” The reference group for the binary “URL link: Yes” variable is “URL link: No.” The date difference variable is a number based on the number of days between the date of data collection and when the Tweet was posted. Results in this section contain DVs that were constructed using the formula *ln*(Y+1).

# **Table S3. Full Preregistered Model Results for Study 1a: Twitter Data**

| DV: Log-transformed Retweets | | | | | |  |
| --- | --- | --- | --- | --- | --- | --- |
| Fixed effects | *B* | *SE* | *df* | *t* | *p* | |
| (Intercept) | 1.12E-01 | 4.99E-03 | 1.47E+03 | 22.47 | < .001 | |
| Numeric tweets | 2.94E-02 | 4.20E-04 | 8.00E+06 | 70.10 | < .001 | |
| Random intercepts | *n* | σ^2^ | *SD* |  |  | |
| Username | 1598 | .039 | .197 |  |  | |
| Total # of observations, *Rm, Rc* | 8003920 | *Rm* = .023 | *Rm* = .371 |  |  | |
|  |  |  |  |  |  | |
|  |  |  |  |  |  | |
| DV: Log-transformed Likes | | | | | |  |
| Fixed effects | *B* | *SE* | *df* | *t* | *p* | |
| (Intercept) | 8.49E-03 | 4.35E-04 | 1.45E+03 | 19.51 | < .001 | |
| Numeric tweets | 3.84E-03 | 7.72E-05 | 8.00E+06 | 49.77 | < .001 | |
| Random intercepts | *n* | σ^2^ | *SD* |  |  | |
| Username | 1598 | .000 | .017 |  |  | |
| Total # of observations, *Rm, Rc* | 8003920 | *Rm* = .018 | *Rm* = .183 |  |  | |
|  |  |  |  |  |  | |
| DV: Engagements index | | | | | |  |
| Fixed effects | *B* | *SE* | *df* | *t* | *p* | |
| (Intercept) | -3.75E-02 | 1.15E-02 | 1.43E+03 | -3.26 | .001 | |
| Numeric tweets | 9.91E-02 | 1.20E-03 | 8.00E+06 | 82.79 | < .001 | |
| Random intercepts | *n* | σ^2^ | *SD* |  |  | |
| Username | 1598 | .205 | .453 |  |  | |
| Total # of observations, *Rm, Rc* | 8003920 | *Rm* = .028 | *Rm* = .307 |  |  | |

# **Table S4. Full Preregistered Model with Reconstructed DV for Study 1a: Twitter Data**

| DV: Log-transformed Retweets | | | | | | | | | |
| --- | --- | --- | --- | --- | --- | --- | --- | --- | --- |
| Fixed effects | *B* | *SE* | *df* | | *t* | | *p* | |  |
| (Intercept) | 1.42E+00 | 1.88E-02 | 1.59E+03 | | 75.52 | | <2e-16 | |  |
| Numeric Tweets | 4.54E-01 | 1.70E-03 | 8.00E+06 | | 266.59 | | <2e-16 | |  |
| Date difference | -2.47E-01 | 8.67E-04 | 7.99E+06 | | -284.54 | | <2e-16 | |  |
| Random intercepts | *n* | σ^2^ | *SD* | |  | |  | |  |
| Username | 1598 | .548 | .741 | |  | |  | |  |
| Total # of observations, *Rm, Rc* | 8003920 | *Rm* = .145 | *Rc* = .372 | |  | |  | |  |
|  |  |  |  | |  | |  | |  |
|  |  |  |  | |  | |  | |  |
| DV: Log-transformed Likes | | | | | | | | | |
| Fixed effects | *B* | *SE* | *df* | *t* | | *p* | |  |  |
| (Intercept) | 4.92E-01 | 7.63E-03 | 1.59E+03 | 64.54 | | <2e-16 | |  |  |
| Numeric Tweets | 5.74E-02 | 7.51E-04 | 8.00E+06 | 76.35 | | <2e-16 | |  |  |
| Date difference | -2.49E-01 | 3.83E-04 | 7.98E+06 | -650.55 | | <2e-16 | |  |  |
| Random intercepts | *n* | σ^2^ | *SD* |  | |  | |  |  |
| Username | 1598 | .090 | .301 |  | |  | |  |  |
| Total # of observations, *Rm, Rc* | 8003920 | *Rm* = .258 | *Rc* = .404 |  | |  | |  |  |
|  |  |  |  |  | |  | |  |  |
|  |  |  |  |  | |  | |  |  |

*Note*. All continuous variables were standardized (z-scored). The reference group for the binary “Numeric tweets” variable is “non-numeric tweets.” The date difference variable is a number based on the number of days between the date of data collection and when the Tweet was posted. Results in this section contain DVs that were constructed using the formula *ln*(Y+1).

# **Table S5. Intercorrelations Among Variables for Study 1a: Twitter**

| Number | Variable | 1 | 2 | 3 | 4 | 5 | 6 | 7 | 8 | 9 |
| --- | --- | --- | --- | --- | --- | --- | --- | --- | --- | --- |
| 1 | Retweets (log) | -- |  |  |  |  |  |  |  |  |
| 2 | Quote tweets (log) | .055 | -- |  |  |  |  |  |  |  |
| 3 | Likes (log) | .042 | .568 | -- |  |  |  |  |  |  |
| 4 | Numeric Tweets | .020 | .011 | .018 | -- |  |  |  |  |  |
| 5 | LIWC Word count | .043 | .035 | .101 | .231 | -- |  |  |  |  |
| 6 | LIWC Emotionality | -.036 | -.004 | -.006 | -.126 | -.207 | -- |  |  |  |
| 7 | LIWC Positivity | -.033 | -.004 | -.005 | -.070 | -.138 | .545 | -- |  |  |
| 8 | Climate words | -.014 | .006 | .006 | -.006 | .009 | -.040 | -.041 | -- |  |
| 9 | LIWC Verbal numeric terms | .019 | .001 | .003 | .022 | .023 | -.038 | -.016 | -.006 | -- |
| 10 | Follower count | -.024 | .028 | .083 | -.034 | -.005 | .028 | -.001 | .017 | -.001 |

*Note*. All correlations are significant at *p* < .01. Engagement metrics were natural log transformed using the formula *ln*(Y+1).

# **Table S6. Full Covariate Model Results for Study 1b: reddit Data**

| DV: Log-transformed Upvotes for Posts | | | | | |
| --- | --- | --- | --- | --- | --- |
| Fixed effects | *B* | *SE* | *df* | *t* | *p* |
| (Intercept) | 3.19E-01 | 1.91E-02 | 7.14E+02 | 16.69 | < .001 |
| Numeric reddit text | 8.31E-02 | 3.77E-02 | 7.93E+02 | 2.20 | .028 |
| LIWC word count | 6.78E-03 | 1.55E-02 | 9.00E+02 | 0.44 | .661 |
| LIWC Emotionality | 3.37E-02 | 1.41E-02 | 8.23E+02 | 2.40 | .017 |
| Climate words | -8.91E-03 | 1.47E-02 | 8.90E+02 | -0.61 | .544 |
| LIWC Verbal numeric terms | 1.96E-02 | 1.36E-02 | 6.64E+02 | 1.44 | .151 |
| Random intercepts | *n* | σ^2^ | *SD* |  |  |
| Author | 661 | .111 | .333 |  |  |
| Total # of observations, *Rm, Rc* | 950 | *Rm* = .108 | *Rc* = .702 |  |  |
|  |  |  |  |  |  |
|  |  |  |  |  |  |
| \| DV: Log-transformed Upvotes for Comments \| \| \| \| \| \| \| \| --- \| --- \| --- \| --- \| --- \| --- \| --- \| \| Fixed effects \| *B* \| *SE* \| *df* \| *t* \| *p* \| \| (Intercept) \| 6.24E-02 \| 4.41E-03 \| 9.45E+02 \| 14.14 \| < .001 \| \| Numeric reddit text \| 2.77E-03 \| 1.87E-03 \| 1.53E+04 \| 1.48 \| .140 \| \| LIWC word count \| 3.38E-03 \| 8.70E-04 \| 1.52E+04 \| 3.88 \| < .001 \| \| LIWC Emotionality \| -1.13E-03 \| 7.98E-04 \| 1.56E+04 \| -1.42 \| .157 \| \| Climate words \| 2.03E-03 \| 7.78E-04 \| 1.55E+04 \| 2.61 \| .009 \| \| LIWC Verbal numeric terms \| 1.27E-03 \| 7.63E-04 \| 1.51E+04 \| 1.67 \| .095 \| \| Contains weblink (URL link: Yes) \| 6.23E-03 \| 2.60E-03 \| 1.56E+04 \| 2.39 \| .017 \| \| Random intercepts \| *n* \| σ^2^ \| *SD* \|  \|  \| \| Author \| 3362 \| .002 \| .041 \|  \|  \| \| Subreddit thread \| 830 \| .013 \| .115 \|  \|  \| \| Total # of observations, *Rm, Rc* \| 16190 \| *Rm* = .038 \| *Rc* = .804 \|  \|  \| \|  \|  \|  \|  \|  \|  \| \|  \|  \|  \|  \|  \|  \|   DV: Log-transformed Upvotes for Posts | | | | | |
| Fixed effects | *B* | *SE* | *df* | *t* | *p* |
| (Intercept) | 3.19E-01 | 1.91E-02 | 7.15E+02 | 16.74 | < .001 |
| Numeric reddit text | 7.85E-02 | 3.76E-02 | 7.94E+02 | 2.09 | .037 |
| LIWC word count | 5.36E-03 | 1.54E-02 | 8.99E+02 | 0.35 | .728 |
| LIWC Positivity | 3.73E-02 | 1.36E-02 | 6.33E+02 | 2.75 | .006 |
| Climate words | -5.48E-03 | 1.47E-02 | 9.02E+02 | -0.37 | .710 |
| LIWC Verbal numeric terms | 1.58E-02 | 1.37E-02 | 6.69E+02 | 1.16 | .247 |
| Random intercepts | *n* | σ^2^ | *SD* |  |  |
| Author | 661 | .111 | .334 |  |  |
| Total # of observations, *Rm, Rc* | 950 | *Rm* = .112 | *Rc* = .704 |  |  |
|  |  |  |  |  |  |
| DV: Log-transformed Upvotes for Comments | | | | | |
| Fixed effects | *B* | *SE* | *df* | *t* | *p* |
| (Intercept) | 6.23E-02 | 4.41E-03 | 9.43E+02 | 14.11 | < .001 |
| Numeric reddit text | 2.93E-03 | 1.87E-03 | 1.53E+04 | 1.57 | .117 |
| LIWC word count | 3.40E-03 | 8.70E-04 | 1.52E+04 | 3.91 | < .001 |
| LIWC Positivity | -7.43E-04 | 7.72E-04 | 1.55E+04 | -0.96 | .336 |
| Climate words | 2.07E-03 | 7.77E-04 | 1.55E+04 | 2.66 | .008 |
| LIWC Verbal numeric terms | 1.30E-03 | 7.63E-04 | 1.51E+04 | 1.71 | .088 |
| Contains weblink (URL link: Yes) | 6.30E-03 | 2.60E-03 | 1.56E+04 | 2.42 | .015 |
| Random intercepts | *n* | σ^2^ | *SD* |  |  |
| Author | 3362 | .002 | .041 |  |  |
| Subreddit thread | 830 | .013 | .115 |  |  |
| Total # of observations, *Rm, Rc* | 16190 | *Rm* = .037 | *Rc* = .804 |  |  |

# **Table S7. Full Covariate Model with Reconstructed DV for Study 1b: reddit Data**

| DV: Log-transformed Upvotes for Posts | | | | | |
| --- | --- | --- | --- | --- | --- |
| Fixed effects | *B* | *SE* | *df* | *t* | *p* |
| (Intercept) | 2.54E+00 | 5.34E-02 | 6.53E+02 | 47.70 | < .001 |
| Numeric reddit text | 7.81E-02 | 1.11E-01 | 8.43E+02 | 0.71 | .481 |
| LIWC word count | 1.03E-01 | 4.50E-02 | 9.30E+02 | 2.29 | .022 |
| LIWC Emotionality | 6.78E-02 | 4.13E-02 | 8.58E+02 | 1.64 | .101 |
| Climate words | -2.71E-02 | 4.27E-02 | 9.17E+02 | -0.64 | .525 |
| LIWC Verbal numeric terms | 1.24E-01 | 4.04E-02 | 7.13E+02 | 3.06 | .002 |
| Date difference | 2.45E-02 | 4.36E-02 | 9.37E+02 | 0.56 | .575 |
| Random intercepts | *n* | σ^2^ | *SD* |  |  |
| Author | 661 | .673 | .820 |  |  |
| Total # of observations, *Rm, Rc* | 950 | *Rm* = .140 | *Rc* = .621 |  |  |
|  |  |  |  |  |  |
|  |  |  |  |  |  |
| \| DV: Log-transformed Upvotes for Comments \| \| \| \| \| \| \| \| --- \| --- \| --- \| --- \| --- \| --- \| --- \| \| Fixed effects \| *B* \| *SE* \| *df* \| *t* \| *p* \| \| (Intercept) \| 1.17E+00 \| 1.25E-02 \| 1.46E+03 \| 93.08 \| < .001 \| \| Numeric reddit text \| 3.58E-02 \| 1.24E-02 \| 1.58E+04 \| 2.89 \| .004 \| \| LIWC word count \| 2.72E-02 \| 5.75E-03 \| 1.57E+04 \| 4.73 \| < .001 \| \| LIWC Emotionality \| -9.12E-03 \| 5.25E-03 \| 1.60E+04 \| -1.74 \| .083 \| \| Climate words \| 2.12E-02 \| 5.13E-03 \| 1.59E+04 \| 4.13 \| < .001 \| \| LIWC Verbal numeric terms \| 1.23E-02 \| 5.08E-03 \| 1.56E+04 \| 2.42 \| .016 \| \| Contains weblink (URL link: Yes) \| 5.88E-02 \| 1.72E-02 \| 1.60E+04 \| 3.42 \| .001 \| \| Date difference \| 2.04E-02 \| 9.21E-03 \| 7.69E+02 \| 2.21 \| .027 \| \| Random intercepts \| *n* \| σ^2^ \| *SD* \|  \|  \| \| Author \| 3362 \| .097 \| .311 \|  \|  \| \| Subreddit thread \| 830 \| .030 \| .174 \|  \|  \| \| Total # of observations, *Rm, Rc* \| 16190 \| *Rm* = .081 \| *Rc* = .511 \|  \|  \| \|  \|  \|  \|  \|  \|  \| \|  \|  \|  \|  \|  \|  \|   DV: Log-transformed Upvotes for Posts | | | | | |
| Fixed effects | *B* | *SE* | *df* | *t* | *p* |
| (Intercept) | 2.55E+00 | 5.35E-02 | 6.55E+02 | 47.63 | < .001 |
| Numeric reddit text | 7.10E-02 | 1.10E-01 | 8.37E+02 | 0.64 | .520 |
| LIWC word count | 1.00E-01 | 4.48E-02 | 9.27E+02 | 2.24 | .026 |
| LIWC Positivity | 8.63E-02 | 4.03E-02 | 6.76E+02 | 2.14 | .033 |
| Climate words | -2.01E-02 | 4.28E-02 | 9.23E+02 | -0.47 | .638 |
| LIWC Verbal numeric terms | 1.15E-01 | 4.05E-02 | 7.08E+02 | 2.83 | .005 |
| Date difference | 2.68E-02 | 4.36E-02 | 9.39E+02 | 0.62 | .539 |
| Random intercepts | *n* | σ^2^ | *SD* |  |  |
| Author | 661 | .695 | .834 |  |  |
| Total # of observations, *Rm, Rc* | 950 | *Rm* = .143 | *Rc* = .630 |  |  |
|  |  |  |  |  |  |
| DV: Log-transformed Upvotes for Comments | | | | | |
| Fixed effects | *B* | *SE* | *df* | *t* | *p* |
| (Intercept) | 1.16E+00 | 1.25E-02 | 1.45E+03 | 93.14 | < .001 |
| Numeric reddit text | 3.74E-02 | 1.24E-02 | 1.58E+04 | 3.03 | .002 |
| LIWC word count | 2.75E-02 | 5.75E-03 | 1.57E+04 | 4.79 | < .001 |
| LIWC Positivity | -2.41E-03 | 5.17E-03 | 1.60E+04 | -0.47 | .642 |
| Climate words | 2.17E-02 | 5.13E-03 | 1.59E+04 | 4.23 | < .001 |
| LIWC Verbal numeric terms | 1.25E-02 | 5.08E-03 | 1.56E+04 | 2.46 | .014 |
| Contains weblink (URL link: Yes) | 5.94E-02 | 1.72E-02 | 1.60E+04 | 3.45 | .001 |
| Date difference | 2.03E-02 | 9.20E-03 | 7.68E+02 | 2.20 | .028 |
| Random intercepts | *n* | σ^2^ | *SD* |  |  |
| Author | 3362 | .097 | .311 |  |  |
| Subreddit thread | 830 | .030 | .174 |  |  |
| Total # of observations, *Rm, Rc* | 16190 | *Rm* = .080 | *Rc* = .511 |  |  |

*Note*. All continuous variables were standardized (z-scored). The reference group for the binary “Numeric reddit text” variable is “non-numeric reddit text.” The date difference variable is a number based on the number of days between the date of data collection and when the reddit data were posted. Results in this section contain DVs that were constructed using the formula *ln*(Y+1).

# **Table S8. Full Preregistered Model Results for Study 1b: reddit Data**

| DV: Log-transformed Upvotes for Posts | | | | | |  |
| --- | --- | --- | --- | --- | --- | --- |
| Fixed effects | *B* | *SE* | *df* | *t* | *p* | |
| (Intercept) | 3.17E-01 | 1.89E-02 | 7.24E+02 | 16.76 | < .001 | |
| Numeric reddit text | 7.70E-02 | 3.56E-02 | 8.25E+02 | 2.16 | .031 | |
| Random intercepts | *n* | σ^2^ | *SD* |  |  | |
| Author | 668 | .110 | .331 |  |  | |
| Total # of observations, *Rm, Rc* | 962 | *Rm* = .064 | *Rc* = .695 |  |  | |
|  |  |  |  |  |  | |
| DV: Log-transformed Upvotes for Comments | | | | | |  |
| Fixed effects | *B* | *SE* | *df* | *t* | *p* | |
| (Intercept) | 6.13E-02 | 4.38E-03 | 9.44E+02 | 13.98 | < .001 | |
| Numeric reddit text | 6.86E-03 | 1.73E-03 | 1.58E+04 | 3.98 | < .001 | |
| Random intercepts | *n* | σ^2^ | *SD* |  |  | |
| Author | 3392 | .002 | .042 |  |  | |
| Subreddit thread | 836 | .013 | .115 |  |  | |
| Total # of observations, *Rm, Rc* | 16539 | *Rm* = .021 | *Rc* = .804 |  |  | |

# **Table S9. Full Preregistered Model with Reconstructed DV for Study 1b: reddit Data**

| DV: Log-transformed Upvotes for Posts | | | | | |
| --- | --- | --- | --- | --- | --- |
| Fixed effects | *B* | *SE* | *df* | *t* | *p* |
| (Intercept) | 2.53E+00 | 5.36E-02 | 6.73E+02 | 47.15 | < .001 |
| Numeric reddit text | 1.41E-01 | 1.06E-01 | 8.77E+02 | 1.33 | .183 |
| Date difference | 2.57E-02 | 4.37E-02 | 9.53E+02 | 0.59 | .557 |
| Random intercepts | *n* | σ^2^ | *SD* |  |  |
| Author | 668 | .678 | .824 |  |  |
| Total # of observations, *Rm, Rc* | 962 | *Rm* = .046 | *Rc* = .607 |  |  |
|  |  |  |  |  |  |
| DV: Log-transformed Upvotes for Comments | | | | | |
| Fixed effects | *B* | *SE* | *df* | *t* | *p* |
| (Intercept) | 1.16E+00 | 1.23E-02 | 1.41E+03 | 94.44 | < .001 |
| Numeric reddit text | 6.76E-02 | 1.14E-02 | 1.63E+04 | 5.91 | < .001 |
| Date difference | 1.99E-02 | 9.13E-03 | 7.68E+02 | 2.18 | .030 |
| Random intercepts | *n* | σ^2^ | *SD* |  |  |
| Author | 3392 | .098 | .313 |  |  |
| Subreddit thread | 836 | .030 | .173 |  |  |
| Total # of observations, *Rm, Rc* | 16539 | *Rm* = .053 | *Rc* = .508 |  |  |

*Note*. All continuous variables were standardized (z-scored). The reference group for the binary “Numeric reddit text” variable is “non-numeric reddit text.” The date difference variable is a number based on the number of days between the date of data collection and when the reddit data were posted. Results in this section contain DVs that were constructed using the formula *ln*(Y+1).

# **Table S10. Intercorrelations Among Variables for Study 1b: reddit**

| Number | Correlations for Posts | | 1 | | 2 | | 3 | | | 4 | 5 | | | 6 | | 7 | |
| --- | --- | --- | --- | --- | --- | --- | --- | --- | --- | --- | --- | --- | --- | --- | --- | --- | --- |
| 1 | Upvotes (log) | | -- | |  | |  | | |  |  | | |  | |  | |
| 2 | Numeric posts | | .065 | | -- | |  | | |  |  | | |  | |  | |
| 3 | LIWC word count | | .038 | | .234 | | -- | | |  |  | | |  | |  | |
| 4 | LIWC Emotionality | | .050 | | -.082 | | -.121 | | | -- |  | | |  | |  | |
| 5 | LIWC Positivity | | .071 | | -.010 | | -.054 | | | .368 | -- | | |  | |  | |
| 6 | Climate words | | -.032 | | -.144 | | -.189 | | | .031 | -.031 | | | -- | |  | |
| 7 | LIWC Verbal numeric terms | | .080 | | .047 | | .078 | | | -.011 | .080 | | | -.022 | | -- | |
| 8 | Contains weblink | | NA | | NA | | NA | | | NA | NA | | | NA | | NA | |
| Number | *P*-values for Posts | | 1 | | 2 | | 3 | | | 4 | 5 | | | 6 | | 7 | |
| 1 | Upvotes (log) | | -- | |  | |  | | |  |  | | |  | |  | |
| 2 | Numeric posts | | .047 | | -- | |  | | |  |  | | |  | |  | |
| 3 | LIWC word count | | .242 | | .000 | | -- | | |  |  | | |  | |  | |
| 4 | LIWC Emotionality | | .123 | | .011 | | .000 | | | -- |  | | |  | |  | |
| 5 | LIWC Positivity | | .028 | | .751 | | .096 | | | .000 | -- | | |  | |  | |
| 6 | Climate words | | .324 | | .000 | | .000 | | | .342 | .337 | | | -- | |  | |
| 7 | LIWC Verbal numeric terms | | .014 | | .147 | | .016 | | | .745 | .013 | | | .508 | | -- | |
| 8 | Contains weblink | | NA | | NA | | NA | | | NA | NA | | | NA | | NA | |
| Number | | Correlation for Comments | | 1 | | 2 | | 3 | 4 | | | 5 | 6 | | 7 | |  |
| 1 | | Upvotes (log) | | -- | |  | |  |  | | |  |  | |  | |  |
| 2 | | Numeric posts | | -.010 | | -- | |  |  | | |  |  | |  | |  |
| 3 | | LIWC word count | | .026 | | .381 | | -- |  | | |  |  | |  | |  |
| 4 | | LIWC Emotionality | | -.010 | | -.142 | | -.104 | -- | | |  |  | |  | |  |
| 5 | | LIWC Positivity | | -.007 | | -.069 | | -.059 | .422 | | | -- |  | |  | |  |
| 6 | | Climate words | | .027 | | -.001 | | .018 | -.065 | | | -.042 | -- | |  | |  |
| 7 | | LIWC Verbal numeric terms | | .016 | | .040 | | .009 | -.028 | | | .004 | -.010 | | -- | |  |
| 8 | | Contains weblink | | .032 | | .177 | | .168 | -.063 | | | -.029 | .007 | | .023 | |  |
| Number | | *P*-values for Comments | | 1 | | 2 | | 3 | 4 | | | 5 | 6 | | 7 | |  |
| 1 | | Upvotes (log) | | -- | |  | |  |  | | |  |  | |  | |  |
| 2 | | Numeric posts | | .183 | | -- | |  |  | | |  |  | |  | |  |
| 3 | | LIWC word count | | .001 | | .000 | | -- |  | | |  |  | |  | |  |
| 4 | | LIWC Emotionality | | .222 | | .000 | | .000 | -- | | |  |  | |  | |  |
| 5 | | LIWC Positivity | | .367 | | .000 | | .000 | .000 | | | -- |  | |  | |  |
| 6 | | Climate words | | .001 | | .863 | | .024 | .000 | | | .000 | -- | |  | |  |
| 7 | | LIWC Verbal numeric terms | | .048 | | .000 | | .279 | .000 | | | .650 | .187 | | -- | |  |
| 8 | | Contains weblink | | .000 | | .000 | | .000 | .000 | | | .000 | .375 | | .004 | |  |

*Note*. Upvotes were natural log transformed using the formula *ln*(Y+1).

# **Table S11. Stimuli in Study 2: Within-Participants Experiment**

All Study 2 Tweets described the same climate consequences whose numeric exactness decreased from one Tweet type to the next. The latter two types had the same low exactness about consequences, and the final Tweet type, Arabic-number non-consequences, contained at least one Arabic number about ancillary information (e.g., the year 2014).

| Arabic-number consequences | Verbal-number consequences | Non-numeric consequences | Arabic-number non-consequences |
| --- | --- | --- | --- |
| A decade ago, 58.4% of California experienced EXCEPTIONAL DROUGHT - the highest recognized category of drought. Even now, 84.6% of California still experiences moderate drought. | A decade ago, more than half of California experienced EXCEPTIONAL DROUGHT - the highest recognized category of drought. Even now, most of California still experiences moderate drought. | A decade ago, much of California experienced EXCEPTIONAL DROUGHT - the highest recognized category of drought. Even now, most of California still experiences moderate drought. | In 2014, much of California experienced EXCEPTIONAL DROUGHT - the highest recognized category of drought. Even now, most of California still experiences moderate drought. |
| According to NASA, the last decade has included the WARMEST years on record, with an average global temperature increase of 1.9 degrees F. | According to NASA, the last decade has included the WARMEST years on record, with an average global temperature increase of a couple of degrees F. | According to NASA, the last decade has included the WARMEST years on record, based on average global temperatures. | According to NASA, the last decade has included the WARMEST years on record, with 2020 and 2016 being tied for the hottest average global temperatures. |
| Over 90% of the fuel used for transportation in the US is petroleum based, and transportation is responsible for 27% of ALL greenhouse gas emissions. | Almost all of the fuel used for transportation in the US is petroleum based, and transportation is responsible for more than a quarter of ALL greenhouse gas emissions. | Almost all of the fuel used for transportation in the US is petroleum based, and transportation is responsible for a lot of the greenhouse gas emissions. | Almost all of the fuel used for transportation in the US is petroleum based, and transportation is responsible for a lot of the greenhouse gas emissions according to 2020 reports. |
| NASA data shows that the Arctic has warmed more than 3 times faster than the rest of the globe. Sea ice has melted by 13% per decade, too. | NASA data shows that the Arctic has warmed more than several times faster than the rest of the globe. Sea ice has melted by multiple percentages per decade, too. | NASA data shows that the Arctic has warmed faster than the rest of the globe. Sea ice has melted each decade, too. | NASA data from 2020 shows that the Arctic has warmed faster than the rest of the globe. Sea ice has melted each decade, too. |
| The US is the LEADING PRODUCER of plastic waste globally at 42 million metric tons. | The US is the LEADING PRODUCER of plastic waste globally, outputting millions of metric tons. | The US is the LEADING PRODUCER of plastic waste globally. | The US is the #1 PRODUCER of plastic waste globally. |
| Global sea levels hit a RECORD HIGH in recent years, at 2.8 inches higher than 3 decades ago. | Global sea levels hit a RECORD HIGH in recent years, at several inches higher than a few decades ago. | Global sea levels hit a RECORD HIGH in recent years compared to a few decades ago. | Global sea levels hit a RECORD HIGH in 2021 compared to 1993 levels. |
| Carbon dioxide levels in the atmosphere hit a NEW HIGH of 414.7 ppm recently, a 45% increase since people started burning fossil fuels. | Carbon dioxide levels in the atmosphere hit a NEW HIGH recently, an increase of almost half again since people started burning fossil fuels. | Carbon dioxide levels in the atmosphere hit a NEW HIGH recently, a marked increase since people started burning fossil fuels. | CO2 levels in the atmosphere hit a NEW HIGH in 2021, a marked increase since people started burning fossil fuels. |
| Extreme weather has been the MOST DAMAGING in the last 5 years, reaching about $152.9 billion per year in repair costs. | Extreme weather has been the MOST DAMAGING in the last few years, reaching billions per year in repair costs. | Extreme weather has been the MOST DAMAGING in the last few years in terms of annual repair costs. | Extreme weather has been the MOST DAMAGING in the last few years in terms of annual repair costs, based on a COP27 report. |
| Reports indicate that the world is losing species at an UNPRECEDENTED rate; 1,000 times greater than any other time in recorded human history. | Reports indicate that the world is losing species at an UNPRECEDENTED rate; many, many times greater than any other time in recorded human history. | Reports indicate that the world is losing species at an UNPRECEDENTED rate compared to any other time in recorded human history. | Reports from 2019 indicate that the world is losing species at an UNPRECEDENTED rate compared to any other time in recorded human history. |
| Climate change makes wildfires worse, costing the US more than $1 billion every year. | Climate change makes wildfires worse, costing the US billions of dollars every year. | Climate change makes wildfires worse, costing the US a lot of money every year. | Climate change makes wildfires worse, costing the US a lot of money every year, especially since 2000. |
| Planting trees is NOT ENOUGH! We would need to plant 1.6 billion hectares of new forests - the equivalent of 5 times the size of India - to achieve "net zero" carbon emissions. | Planting trees is NOT ENOUGH! We would need to plant billions of hectares of new forests - the equivalent of many times the size of India - to achieve "net zero" carbon emissions. | Planting trees is NOT ENOUGH! We would need to plant huge areas of new forests to achieve "net zero" carbon emissions. | Planting trees is NOT ENOUGH! We would need to plant huge areas of new forests to achieve "net zero" carbon emissions by 2050. |
| Each year, Antarctica loses 151 billion tons of ice. | Each year, Antarctica loses many billions of tons of ice. | Each year, Antarctica loses a lot of ice. | Each year, Antarctica loses a lot of ice, according to COP27. |
| 190 million people are projected to LOSE THEIR HOMES to rising sea levels this century, even under low carbon emissions. | Millions of people are projected to LOSE THEIR HOMES to rising sea levels this century, even under low carbon emissions. | Many people are projected to LOSE THEIR HOMES to rising sea levels this century, even under low carbon emissions. | Many people are projected to LOSE THEIR HOMES to rising sea levels by 2100, even under low carbon emissions. |
| Approximately 95% of our calories from food come from soil, yet topsoil erosion has accelerated by 10-fold due to human activity. | Almost all of our calories from food come from soil, yet topsoil erosion has accelerated by many times due to human activity. | Most of our calories from food come from soil, yet topsoil erosion has accelerated due to human activity. | Most of our calories from food come from soil, yet topsoil erosion has accelerated due to human activity, according to 2019 reports. |
| Heat kills! Heat related mortality has increased by 53.7% for older people. | Heat kills! Heat related mortality has increased by more than half for older people. | Heat kills! Heat related mortality has increased for older people. | Heat kills! Since 2000, heat related mortality has increased for older people. |
| Even with cooler weather due to La Niña conditions, 91% of the Great Barrier Reefs were harmed by coral bleaching. | Even with cooler weather due to La Niña conditions, almost all of the Great Barrier Reefs were harmed by coral bleaching. | Even with cooler weather due to La Niña conditions, the Great Barrier Reefs were harmed by coral bleaching. | Even with cooler weather due to La Niña conditions in 2022, the Great Barrier Reefs were harmed by coral bleaching. |
| Deforestation needs to STOP! 4.7 million hectares of forests are chopped down each year. | Deforestation needs to STOP! Millions of hectares of forests are chopped down each year. | Deforestation needs to STOP! Entire forests are chopped down each year. | Deforestation needs to STOP! Since 2010, entire forests have been chopped down each year. |
| Yet ANOTHER effect of warming temperatures - malaria season has gotten longer by 31.3% in the Americas. | Yet ANOTHER effect of warming temperatures - malaria season has gotten longer by about a third in the Americas. | Yet ANOTHER effect of warming temperatures - malaria season has gotten longer in the Americas. | Yet ANOTHER effect of warming temperatures - malaria season has gotten longer in the Americas, according to a 2021 report. |
| T-shirt weather in Antarctica?! In a recent winter, temperatures soared to a record high of 64.9 degrees F! | T-shirt weather in Antarctica?! In a recent winter, temperatures soared to a record high of almost 70 degrees F! | T-shirt weather in Antarctica?! In a recent winter, temperatures soared to a record high! | T-shirt weather in Antarctica?! In February 2020, temperatures soared to a record high! |
| We lost 295 billion work hours due to heat in one recent year alone; climate change destroys global economies! | We lost billions of work hours due to heat in one recent year alone; climate change destroys global economies! | We lost many work hours to heat in one recent year alone, climate change destroys global economies! | We lost many work hours due to heat in 2020 alone; climate change destroys global economies! |

# **Table S12. Full Covariate Model Results for Study 2: Within-Participants Experiment**

Results (unstandardized b-coefficients (SE) [CI], p-values) of hypotheses concerning fixed effects in mixed model effects with covariates. Each Tweet type was compared to the Arabic-number-consequences Tweet type. p<0.001 is indicated as 0.001.

|  | Likely to share | Want to find out more | Feelings | Came from expert | Trustworthy | Accurate | Clear | Interesting |
| --- | --- | --- | --- | --- | --- | --- | --- | --- |
| Intercept | 2.27 (0.08)  [2.12, 2.43],  0.001 | 2.97 (0.09)  [2.80, 3.13],  0.001 | 2.34 (0.08)  [2.18, 2.50],  0.001 | 3.16 (0.08)  [3.01, 3.31],  0.001 | 4.17 (0.10)  [3.97, 4.37],  0.001 | 4.15 (0.11)  [3.93, 4.36],  0.001 | 4.55 (0.10)  [4.35, 4.74],  0.001 | 4.24 (0.10)  [4.06, 4.43],  0.001 |
| Verbal-number consequences | -0.11 (0.03)  [-0.13, -0.05],  0.001 | -0.11 (0.04)  [-0.18, -0.03],  0.006 | -0.02 (0.04)  [-0.09, 0.05],  0.602 | -0.49 (0.04)  [-0.57, -0.41],  0.001 | -0.21 (0.04)  [-0.30, -0.13],  0.001 | -0.20 (0.05)  [-0.29, -0.11],  0.001 | -0.19 (0.05)  [-0.28, -0.10],  0.001 | -0.11 (0.05)  [-0.20, -0.02],  0.014 |
| Non-numeric consequences | -0.12 (0.04)  [-0.19, -0.05],  0.001 | -0.14 (0.04)  [-0.22, -0.06],  0.001 | 0.08 (0.04)  [0.00, 0.15],  0.044 | -0.55 (0.05)  [-0.64, -0.46],  0.001 | -0.17 (0.05)  [-0.26, -0.07],  0.001 | -0.19 (0.05)  [-0.29, -0.09],  0.001 | -0.22 (0.05)  [-0.32, -0.12],  0.001 | -0.24 (0.05)  [-0.34, -0.15],  0.001 |
| Arabic-number non-consequences | -0.09 (0.03)  [-0.15, -0.02],  0.011 | -0.11 (0.04)  [-0.19, -0.04],  0.004 | 0.06 (0.04)  [-0.01, 0.13],  0.092 | -0.37 (0.04)  [-0.45, -0.29],  0.001 | -0.11 (0.04)  [-0.19, -0.02],  0.016 | -0.09 (0.05)  [-0.18, 0.00],  0.045 | -0.11 (0.05)  [-0.20, -0.02],  0.015 | -0.18 (0.05)  [-0.27, -0.09],  0.001 |
| LIWC Word count | 0.04 (0.03)  [-0.02, 0.10],  0.175 | 0.00 (0.03)  [-0.06, 0.06],  0.959 | 0.04 (0.03)  [-0.03, 0.10],  0.292 | 0.09 (0.04)  [0.01, 0.18],  0.031 | 0.05 (0.04)  [-0.03, 0.14],  0.229 | 0.02 (0.05)  [-0.07, 0.11],  0.704 | -0.02 (0.05)  [-0.11, 0.07],  0.683 | -0.01 (0.04)  [-0.09, 0.08],  0.901 |
| LIWC Emotionality | -0.03 (0.04)  [-0.1, 0.04],  0.353 | -0.04 (0.04)  [-0.11, 0.04],  0.327 | 0.03 (0.05)  [-0.06, 0.12],  0.536 | -0.05 (0.06)  [-0.17, 0.06],  0.395 | -0.03 (0.06)  [-0.15, 0.09],  0.581 | 0.01 (0.07)  [-0.12, 0.15],  0.846 | -0.07 (0.06)  [-0.19, 0.05],  0.257 | -0.06 (0.05)  [-0.16, 0.04],  0.228 |
| Objective Numeracy | -0.26 (0.07)  [-0.40, -0.13],  0.001 | -0.12 (0.07)  [-0.26, 0.02],  0.099 | -0.14 (0.07)  [-0.27, -0.01],  0.034 | -0.06 (0.04)  [-0.15, 0.02],  0.128 | 0.03 (0.08)  [-0.12, 0.18],  0.674 | 0.02 (0.08)  [-0.13, 0.18],  0.754 | 0.06 (0.07)  [-0.08, 0.20],  0.392 | -0.03 (0.08)  [-0.18, 0.12],  0.706 |
|  |  |  |  |  |  |  |  |  |
| Nakagawa Rm (fixed effects) | 0.215 | 0.102 | 0.120 | 0.227 | 0.075 | 0.057 | 0.085 | 0.076 |
| Nakagawa Rc (entire model) | 0.810 | 0.782 | 0.785 | 0.593 | 0.755 | 0.755 | 0.717 | 0.749 |

# **Table S13. Estimated marginal means by Condition for Study 2**

Estimated marginal means (SE) by Tweet type for all primary and secondary dependent measures with covariates (N=212). Continuous variables were set to their mean values. Columns with different letters differed significantly based on Tukey’s adjusted p value<.05. See Table S11 for each comparison.

|  | Arabic-number consequences  (Tweet *n*=1069) | Verbal-number consequences (Tweet *n*=1061) | Non-numeric consequences  (Tweet *n*=1058) | Arabic-number non-consequences  (Tweet *n*=1052) |
| --- | --- | --- | --- | --- |
| **Engagement variables** | | | | |
| Likely to share (1-5) | 2.274 (0.079)^a^ | 2.163 (0.079)^b^ | 2.154 (0.079)^b^ | 2.189 (0.079) |
| Want to find out more (1-5) | 2.966 (0.085)^a^ | 2.861 (0.085)^b^ | 2.823 (0.086)^b^ | 2.856 (0.085)^b^ |
| Feelings (1-5) | 2.337 (0.083) | 2.319 (0.084) | 2.414 (0.084) | 2.396 (0.083) |
| **Opinions about Tweet** | | | | |
| Came from expert (1-5) | 3.157 (0.079)^a^ | 2.669 (0.079)^b^ | 2.603 (0.080)^b^ | 2.785 (0.079)^c^ |
| Trustworthy (1-6) | 4.170 (0.102)^a^ | 3.959 (0.102)^b^ | 4.005 (0.103)^b^ | 4.065 (0.101) |
| Exploratory analyses of Tweet opinions | | | | |
| Accurate (1-6) | 4.145 (0.109)^a^ | 3.948 (0.110)^b^ | 3.952 (0.110)^b^ | 4.053 (0.109) |
| Clear (1-6) | 4.546 (0.100)^a^ | 4.360 (0.101)^b^ | 4.331 (0.101)^b^ | 4.434 (0.100) |
| Interesting (1-6) | 4.244 (0.096)^a^ | 4.134 (0.096)^a,c^ | 4.000 (0.097)^b^ | 4.067 (0.096)^b,c^ |

# **Table S14. Full Results for Condition Comparisons for Study 2**

For each primary, secondary, and exploratory variable (with covariates), estimated marginal means (at mean levels of each continuous variable in the model), standard errors (SE), df, and confidence intervals in each Tweet condition and all contrasts between them including mean difference, SE, df, t-values, Tukey-adjusted p-values, confidence intervals, and effect size indicator (Cohen’s D).

| **Engagement variables** | |  |  |  |  |  |  |  |  |
| --- | --- | --- | --- | --- | --- | --- | --- | --- | --- |
| Likely to share (1-5) | Conditions | Mean | SE | df | lower.CL | upper.CL |  |  |  |
|  | Arabic-number consequences (1) | 2.274 | 0.079 | 194.880 | 2.119 | 2.429 |  |  |  |
|  | Verbal-number consequences (2) | 2.163 | 0.079 | 194.570 | 2.007 | 2.318 |  |  |  |
|  | Non-numeric consequences (3) | 2.154 | 0.079 | 194.050 | 1.997 | 2.310 |  |  |  |
|  | Arabic-number non-consequences (4) | 2.189 | 0.079 | 195.500 | 2.034 | 2.344 |  |  |  |
|  | contrast | Mean Diff | SE | df | t.ratio | p.value | lower.CL | upper.CL | Cohen's D |
|  | 1-2 | 0.112 | 0.033 | 4030.090 | 3.350 | 0.005 | 0.026 | 0.197 | 0.150 |
|  | 1-3 | 0.120 | 0.036 | 1739.910 | 3.325 | 0.005 | 0.027 | 0.213 | 0.162 |
|  | 1-4 | 0.085 | 0.033 | 4013.530 | 2.556 | 0.052 | 0.000 | 0.171 | 0.115 |
|  | 2-3 | 0.009 | 0.038 | 1019.470 | 0.230 | 0.996 | -0.089 | 0.107 | 0.012 |
|  | 2-4 | -0.026 | 0.034 | 3487.660 | -0.762 | 0.871 | -0.114 | 0.062 | -0.035 |
|  | 3-4 | 0.035 | 0.035 | 3090.300 | 1.005 | 0.747 | -0.054 | 0.124 | 0.047 |
| Want to find out more (1-5) | Conditions | Mean | SE | df | lower.CL | upper.CL |  |  |  |
|  | Arabic-number consequences (1) | 2.966 | 0.085 | 191.710 | 2.799 | 3.134 |  |  |  |
|  | Verbal-number consequences (2) | 2.861 | 0.085 | 191.200 | 2.693 | 3.030 |  |  |  |
|  | Non-numeric consequences (3) | 2.823 | 0.086 | 190.430 | 2.654 | 2.992 |  |  |  |
|  | Arabic-number non-consequences (4) | 2.856 | 0.085 | 192.440 | 2.688 | 3.023 |  |  |  |
|  | contrast | Mean Diff | SE | df | t.ratio | p.value | lower.CL | upper.CL | Cohen's D |
|  | 1-2 | 0.105 | 0.038 | 4031.960 | 2.760 | 0.030 | 0.007 | 0.203 | 0.124 |
|  | 1-3 | 0.143 | 0.041 | 1736.650 | 3.481 | 0.003 | 0.037 | 0.249 | 0.169 |
|  | 1-4 | 0.111 | 0.038 | 4015.540 | 2.899 | 0.020 | 0.013 | 0.209 | 0.130 |
|  | 2-3 | 0.038 | 0.043 | 1012.110 | 0.890 | 0.810 | -0.073 | 0.150 | 0.045 |
|  | 2-4 | 0.006 | 0.039 | 3491.370 | 0.146 | 0.999 | -0.095 | 0.106 | 0.007 |
|  | 3-4 | 0.033 | 0.040 | 3093.130 | 0.828 | 0.841 | -0.069 | 0.134 | 0.039 |
| Feel (1-5) | Conditions | Mean | SE | df | lower.CL | upper.CL |  |  |  |
|  | Arabic-number consequences (1) | 2.337 | 0.083 | 124.760 | 2.172 | 2.502 |  |  |  |
|  | Verbal-number consequences (2) | 2.319 | 0.084 | 125.060 | 2.153 | 2.485 |  |  |  |
|  | Non-numeric consequences (3) | 2.414 | 0.084 | 125.360 | 2.247 | 2.581 |  |  |  |
|  | Arabic-number non-consequences (4) | 2.396 | 0.083 | 124.990 | 2.231 | 2.561 |  |  |  |
|  | contrast | Mean Diff | SE | df | t.ratio | p.value | lower.CL | upper.CL | Cohen's D |
|  | 1-2 | 0.018 | 0.035 | 4033.600 | 0.521 | 0.954 | -0.071 | 0.107 | 0.02341725 |
|  | 1-3 | -0.077 | 0.038 | 1911.300 | -2.001 | 0.188 | -0.176 | 0.022 | -0.0995245 |
|  | 1-4 | -0.059 | 0.035 | 4018.660 | -1.684 | 0.332 | -0.149 | 0.031 | -0.0760087 |
|  | 2-3 | -0.095 | 0.041 | 1184.980 | -2.333 | 0.091 | -0.200 | 0.010 | -0.1229417 |
|  | 2-4 | -0.077 | 0.036 | 3550.820 | -2.140 | 0.141 | -0.169 | 0.015 | -0.099426 |
|  | 3-4 | -0.018 | 0.037 | 3191.620 | -0.498 | 0.960 | -0.112 | 0.076 | -0.0235157 |
| **Opinions about Tweet** | |  |  |  |  |  |  |  |  |
| Came from expert (1-5) | Conditions | Mean | SE | df | lower.CL | upper.CL |  |  |  |
|  | Arabic-number consequences (1) | 3.157 | 0.079 | 40.530 | 2.998 | 3.316 |  |  |  |
|  | Verbal-number consequences (2) | 2.669 | 0.079 | 41.170 | 2.509 | 2.830 |  |  |  |
|  | Non-numeric consequences (3) | 2.603 | 0.080 | 41.960 | 2.441 | 2.764 |  |  |  |
|  | Arabic-number non-consequences (4) | 2.785 | 0.079 | 40.500 | 2.626 | 2.944 |  |  |  |
|  | contrast | Mean Diff | SE | df | t.ratio | p.value | lower.CL | upper.CL | Cohen's D |
|  | 1-2 | 0.488 | 0.042 | 4067.620 | 11.745 | <0.001 | 0.381 | 0.594 | 0.527 |
|  | 1-3 | 0.554 | 0.046 | 2071.350 | 11.974 | <0.001 | 0.435 | 0.673 | 0.599 |
|  | 1-4 | 0.372 | 0.042 | 4052.310 | 8.924 | <0.001 | 0.265 | 0.480 | 0.402 |
|  | 2-3 | 0.067 | 0.049 | 1332.650 | 1.357 | 0.527 | -0.060 | 0.193 | 0.072 |
|  | 2-4 | -0.115 | 0.043 | 3637.320 | -2.680 | 0.037 | -0.226 | -0.005 | -0.125 |
|  | 3-4 | 0.182 | 0.044 | 3303.540 | 4.159 | 0.000 | 0.070 | 0.295 | 0.197 |
| Trustworthy (1-6) | Conditions | Mean | SE | df | lower.CL | upper.CL |  |  |  |
|  | Arabic-number consequences (1) | 4.170 | 0.102 | 90.210 | 3.968 | 4.372 |  |  |  |
|  | Verbal-number consequences (2) | 3.959 | 0.102 | 90.750 | 3.757 | 4.162 |  |  |  |
|  | Non-numeric consequences (3) | 4.005 | 0.103 | 91.350 | 3.801 | 4.209 |  |  |  |
|  | Arabic-number non-consequences (4) | 4.065 | 0.101 | 90.290 | 3.863 | 4.267 |  |  |  |
|  | contrast | Mean Diff | SE | df | t.ratio | p.value | lower.CL | upper.CL | Cohen's D |
|  | 1-2 | 0.211 | 0.043 | 4038.030 | 4.865 | <0.001 | 0.099 | 0.322 | 0.219 |
|  | 1-3 | 0.165 | 0.048 | 2039.860 | 3.427 | 0.004 | 0.041 | 0.289 | 0.172 |
|  | 1-4 | 0.105 | 0.044 | 4024.720 | 2.417 | 0.074 | -0.007 | 0.217 | 0.109 |
|  | 2-3 | -0.045 | 0.051 | 1303.840 | -0.887 | 0.812 | -0.177 | 0.086 | -0.047 |
|  | 2-4 | -0.106 | 0.045 | 3603.770 | -2.352 | 0.087 | -0.221 | 0.010 | -0.110 |
|  | 3-4 | 0.060 | 0.046 | 3272.630 | 1.317 | 0.552 | -0.057 | 0.177 | 0.062 |
| **Exploratory analyses of Tweet opinions** | |  |  |  |  |  |  |  |  |
| Accurate (1-6) | Conditions | Mean | SE | df | lower.CL | upper.CL |  |  |  |
|  | Arabic-number consequences (1) | 4.145 | 0.109 | 79.850 | 3.928 | 4.362 |  |  |  |
|  | Verbal-number consequences (2) | 3.948 | 0.110 | 80.460 | 3.730 | 4.166 |  |  |  |
|  | Non-numeric consequences (3) | 3.952 | 0.110 | 81.160 | 3.733 | 4.172 |  |  |  |
|  | Arabic-number non-consequences (4) | 4.053 | 0.109 | 79.880 | 3.837 | 4.270 |  |  |  |
|  | contrast | Mean Diff | SE | df | t.ratio | p.value | lower.CL | upper.CL | Cohen's D |
|  | 1-2 | 0.197 | 0.045 | 4039.020 | 4.344 | 0.000 | 0.080 | 0.313 | 0.195 |
|  | 1-3 | 0.192 | 0.051 | 2152.290 | 3.805 | 0.001 | 0.062 | 0.322 | 0.191 |
|  | 1-4 | 0.091 | 0.045 | 4027.300 | 2.004 | 0.187 | -0.026 | 0.208 | 0.091 |
|  | 2-3 | -0.004 | 0.054 | 1410.390 | -0.076 | 1.000 | -0.143 | 0.134 | -0.004 |
|  | 2-4 | -0.105 | 0.047 | 3645.780 | -2.246 | 0.111 | -0.226 | 0.015 | -0.105 |
|  | 3-4 | 0.101 | 0.048 | 3339.360 | 2.122 | 0.146 | -0.021 | 0.224 | 0.101 |
| Clear (1-6) | Conditions | Mean | SE | df | lower.CL | upper.CL |  |  |  |
|  | Arabic-number consequences (1) | 4.546 | 0.100 | 81.340 | 4.347 | 4.745 |  |  |  |
|  | Verbal-number consequences (2) | 4.360 | 0.101 | 81.860 | 4.160 | 4.560 |  |  |  |
|  | Non-numeric consequences (3) | 4.331 | 0.101 | 82.450 | 4.130 | 4.533 |  |  |  |
|  | Arabic-number non-consequences (4) | 4.434 | 0.100 | 81.440 | 4.235 | 4.633 |  |  |  |
|  | contrast | Mean Diff | SE | df | t.ratio | p.value | lower.CL | upper.CL | Cohen's D |
|  | 1-2 | 0.186 | 0.046 | 4041.900 | 4.039 | 0.000 | 0.068 | 0.305 | 0.181 |
|  | 1-3 | 0.215 | 0.051 | 1972.570 | 4.204 | 0.000 | 0.084 | 0.347 | 0.210 |
|  | 1-4 | 0.113 | 0.046 | 4027.260 | 2.430 | 0.072 | -0.006 | 0.232 | 0.110 |
|  | 2-3 | 0.029 | 0.054 | 1241.290 | 0.533 | 0.951 | -0.111 | 0.169 | 0.028 |
|  | 2-4 | -0.074 | 0.048 | 3580.920 | -1.543 | 0.412 | -0.196 | 0.049 | -0.072 |
|  | 3-4 | 0.103 | 0.049 | 3233.280 | 2.115 | 0.148 | -0.022 | 0.227 | 0.100 |
| Interesting (1-6) | Conditions | Mean | SE | df | lower.CL | upper.CL |  |  |  |
|  | Arabic-number consequences (1) | 4.244 | 0.096 | 140.440 | 4.054 | 4.433 |  |  |  |
|  | Verbal-number consequences (2) | 4.134 | 0.096 | 140.280 | 3.944 | 4.325 |  |  |  |
|  | Non-numeric consequences (3) | 4.000 | 0.097 | 139.980 | 3.809 | 4.191 |  |  |  |
|  | Arabic-number non-consequences (4) | 4.067 | 0.096 | 140.950 | 3.877 | 4.256 |  |  |  |
|  | contrast | Mean Diff | SE | df | t.ratio | p.value | lower.CL | upper.CL | Cohen's D |
|  | 1-2 | 0.109 | 0.045 | 4035.410 | 2.447 | 0.069 | -0.006 | 0.224 | 0.110 |
|  | 1-3 | 0.243 | 0.049 | 1771.170 | 4.986 | <0.001 | 0.118 | 0.369 | 0.245 |
|  | 1-4 | 0.177 | 0.045 | 4018.440 | 3.950 | 0.001 | 0.062 | 0.292 | 0.178 |
|  | 2-3 | 0.134 | 0.051 | 1054.180 | 2.610 | 0.045 | 0.002 | 0.267 | 0.135 |
|  | 2-4 | 0.068 | 0.046 | 3498.790 | 1.476 | 0.452 | -0.050 | 0.186 | 0.068 |
|  | 3-4 | 0.066 | 0.047 | 3106.440 | 1.424 | 0.484 | -0.053 | 0.186 | 0.067 |

# **Supplemental Text 1. Exploratory Mediation Analyses for Study 2**

We also explored whether perceived trust and expertise mediated the results of Arabic-number-consequence information (vs the other three conditions) on likelihood to share, wanting to find out more, and feelings in three independent tests. Although methods exist to test for mediation in within-participant designs (3, 4), they do not allow for the inclusion of covariates or for mediators and moderators in a single model. Therefore, we fall back on the classic convention (5) that mediation can be inferred (a) when a condition has an effect on an hypothesized dependent variable and hypothesized mediators, and (b) when the condition effect on the dependent variable is reduced to non-significance when the proposed mediator(s) is included in the model. We acknowledge that this approach has issues (6); thus, the following analyses should be interpreted cautiously, as suggestive of mediation vs. indicative of mediation.

Using mixed models in SPSS, we conducted a series of exploratory models. As in the main text, we used fixed effects and controlled for word count, emotionality of the tweet, and objective numeracy of the individual participant. Then, in the “mediation” models, we added perceptions of trustworthiness and expertise. For each outcome, we conducted a model paralleling the results in Table S12 (i.e., condition, word count, emotionality of the tweet, and objective numeracy as predictors). Then, we used a model with the proposed mediators (i.e., trustworthiness and expertise) replacing condition. Finally, we included condition and the mediators in the same model (with covariates). Mediation is more likely if the effect of condition falls to non-significance when the mediators are included in the model. We omit tests of condition on mediators here and effects of covariates on the outcomes, as they are already reported elsewhere in the manuscript and exploratory models.

First, we confirmed significant effects of condition for sharing; as in the main text and other analyses reported here, a significant effect of condition emerged, F(3,4170.23)=3.92, p=.008. In a second model, sharing was significantly predicted by both opinions of trustworthiness and expertise, F(1,4030.28)=565.94, p<.001 and F(1,40834.92)=131.93, p<.001, respectively. When opinions of trustworthiness and expertise were added to the model, both had significant effects on sharing F(1,4030.80)=565.65, p<.001 and F(1,4089.64)=127.53, p<.001, respectively. The effect of condition was eliminated (F<1).

Second, for interest in finding out more, the effect of condition once again emerged, F(3,4201.61)=4.02, p=.007. In a second model, interest in finding out more was significantly predicted by both opinions of trustworthiness and expertise, F(1,4118.96)=551.66, p<.001 and F(1,4126.29)=60.86, p<.001, respectively. When opinions of trustworthiness and expertise were added to the model, both had significant effects on finding out more, F(1,4119.31)=551.79, p<.001 and F(1,4132.34)=57.06, p<.001, respectively. The effect of condition was eliminated (F<1).

Finally, we confirmed significant effects of condition for feeling (our measure of Twitter “likes”), F(3,4107.12)=5.85, p<.001. In a second model, feelings were also influenced by opinions of trustworthiness and expertise, F(1,4098.13)=64.68, p<.001 and F(1,4121.35)=13.67, p<.001. When opinions of trustworthiness and expertise were added to the same model as condition, the effects of all three variables were significant unlike the models for sharing and finding out more: F_trustworthy_(1,4095.84)=62.28, p<.001, F_expert_(1,4121.12)=10.66, p=.001, F_condition_(3,4099.40)=5.34, p=.001.

Thus, for sharing and finding out more, the results suggest that the effects of condition were moderated by opinions that the source was trustworthy and expert. In other words, numbers about consequences increased perceived trustworthiness and expertise, which in turn increased intentions to share and interest in finding out more. For feelings, however, the effects of condition, expertise, and trustworthiness all were independent. As mentioned in the main text, we reasoned that “likes,” more than sharing, may be more complex than simple engagement and also reflect negative feelings about message content and the dismaying magnitude of climate threats. In other words, the presence of numbers about climate consequences may make these consequences (all of which were negative) seem worse.

# **Supplemental Text 2. Moderator analyses for Study 2: Within-Participant Tweet Experiment**

In the baseline session, we assessed individual differences.

*Objective numeracy* was assessed via an adaptive numeracy test. Participants were asked to solve four math word problems (e.g., If the probability of getting the common cold is 60% in 1 year, what is the probability of getting the common cold in 2 years?) that became harder or easier based on their performance. Each participant earned a score from 0 to 9.

*Number preferences* (28) was measured using the averaged response to four questions on six-point scales asking about number preferences and usefulness (Cronbach’s alpha=.82).

*Political ideology* was self-reported, “Which of the following best describes your political views in general?” on a five-point scale (1=very conservative to 5=very liberal).

Additional exploratory moderator analyses substituting climate-change beliefs or affect for ideology were substantially similar.

*Beliefs in climate change* were assessed by asking participants to chose the statement that best described their view (1=not happening, 2=happening and is caused primarily by natural patterns, and 3=happening and is caused primarily by human activities).

*Baseline affect* was assessed with the HUE (7), in which participants indicate whether they feel various discrete emotions when thinking about climate change (1=does not apply/describe; 5=completely describes). The discrete emotions were afraid, worried, sad, upset, angry, disgusted, and guilty (Cronbach’s α = .92).

Table S15 provides full results for moderator analyses.

*Objective numeracy.* The highly numerate shared all Tweet types less than the less numerate, b=-0.275, SE=0.07, p<.001. Their lower engagement may have been driven partly by lower perceptions that Tweets other than Arabic-number-consequences Tweets came from experts, b_VerbalC:ArabicC_=-0.063, SE=0.043, p=.141, b_NonnumC:ArabicC_=-0.088, SE=0.043, p=.043, b_ArabicNC:ArabicC_=-0.087, SE=0.042, p=.040 (Fig S1a). The highly numerate also had more negative feelings (b=-0.182, SE=.072, p=.013) and especially about non-numeric-consequences and Arabic-number-non-consequences tweets vs Arabic-number-consequence Tweets, b_NonnumC:ArabicC_=0.094, SE=0.036 p=.009 and b_ArabicNC:ArabicC_=0.078, SE=0.036, p=.029; however, they rated Arabic-number-consequences Tweets as more interesting *vs* non-numeric-consequence Tweets, b_NonnumC:ArabicC_=-0.100, SE=0.046 p=.032.

*Number preferences.* Responses on this measure generally were unrelated to Tweet engagement; people with higher *vs* lower preferences reported feeling and sharing similarly although those with greater number preferences wanted to find out more (b=0.152, SE=0.073, p=.049). Number preferences related instead to perceptions of the message source and content. For example, those higher in number preferences perceived Tweets as more interesting (b=0.328, SE=0.078 p<.001) and likely from an expert, p=0.138, SE=0.05 p=.006 (especially Arabic-number-consequences *vs* non-numeric-consequences Tweets, b_NonnumC:ArabicC_=-0.093, SE=0.044, p=.033; Fig S1b). Similar results emerged for perceived trust and other opinion questions such as accurate, clear, and interesting.

*Political ideology*. Liberals were more likely to share (b=0.261, SE=0.070, p<.001) (Fig S2 in the main text), wanted to find out more (b=.322, SE=0.074, p<.001), and found the Tweets trustworthy, interesting, clear, and accurate; of surprise, ideology was unrelated to positive vs negative feelings about the Tweets. Tweet type affected conservatives less than liberals although conservatives did perceive Arabic-number-consequences Tweets as more likely from an expert, b_VerbalC:ArabicC_=-0.034, SE=0.042, p=.416, b_NonnumC:ArabicC_=-0.114, SE=0.041, p=.005, b_ArabicNC:ArabicC_=-0.080, SE=0.041, p=.052 (Fig S1c).

Unsurprisingly, ideology correlated with baseline beliefs in climate change (r=.51) and with affect towards climate change (r=.49). Because of these high correlations with ideology and with each other (r=.56), we conducted exploratory analyses using the same moderators, but replaced ideology, in turn, with belief in climate change and then climate change affect.

*Belief in climate change*. Paralleling the results with ideology, people who believed more in climate change were more likely to share Tweets, b=0.363, SE=0.068, p<.001, and especially Arabic-number-consequences Tweets relative to non-numeric Tweets, b_NonnumC:ArabicC_=-0.071, SE= 0.034, p=.037. They also were more interested in finding out more, b=0.439, SE=0.071, p<.001, and rated tweets as more trustworthy, b=0.641, SE=0.065, p<.001, accurate, b=0.682, SE=0.068, p<.001, clear, b=0.524, SE=0.067, p<.001, and interesting, b=0.589, SE=0.069, p<.001. Those who believed more in climate change also perceived Arabic-number-consequences Tweets as more likely from an expert, b_VerbalC:ArabicC_=-0.034, SE=0.042, p=.414, b_NonnumC:ArabicC_=-0.124, SE= 0.042, p=.003, b_ArabicNC:ArabicC_=-0.136, SE=0.041, p<.001. Those who believed less in climate change had more negative feelings to Arabic-number-non-consequence Tweets, b_ArabicNC:ArabicC_=-0.101, SE=0.034, p=.003.

*Affect towards climate change*. Affect had the same effects as beliefs and overall ideology. Those with more negative affect to climate change were more likely to share, b=0.434, SE=0.065, p<.001 and want to find out more, b=0.583, SE=0.066, p<.001. They also viewed Tweets as more likely to come from an expert, b=0.229, SE=0.046, p<.001, trustworthy, b=0.549, SE=0.068, p<.001, accurate, b=0.578, SE=0.080, p<.001, clear, b=0.407, SE=0.070, p<.001, and interesting, b=0.635, SE=0.68, p<.001.

# **Table S15. Full Moderator Model Results for Study 2: Within-Participants Experiment**

Inferential statistics (b-coefficients (SE) [CI], p-values) of moderators of Tweet type effects. Each Tweet type was compared to the Arabic-numeric-consequences Tweet type. p<0.001 is indicated as 0.001.

|  | Likely to share | Want to find out more | Feelings | Came from expert | Trustworthy | Accurate | Clear | Interesting |
| --- | --- | --- | --- | --- | --- | --- | --- | --- |
| (Intercept) | 2.27 (0.08) [2.12, 2.42], 0.001 | 2.97 (0.08) [2.81, 3.13], 0.001 | 2.34 (0.08) [2.18, 2.50], 0.001 | 3.16 (0.08) [3.00, 3.31], 0.001 | 4.17 (0.09) [3.99, 4.35], 0.001 | 4.144 (0.101) [3.948, 4.34], 0.001 | 4.55 (0.10) [4.36, 4.73], 0.001 | 4.24 (0.09)  [4.07, 4.42], 0.001 |
| Verbal-number consequences | -0.11 (0.03)  [-0.18, -0.05], 0.001 | -0.11 (0.04)  [-0.18, -0.03], 0.006 | -0.02 (0.04)  [-0.09, 0.05], 0.624 | -0.49 (0.04)  [-0.57, -0.41], 0.001 | -0.21 (0.04)  [-0.30, -0.13], 0.001 | -0.198 (0.045)  [-0.29, -0.11], 0.001 | -0.19 (0.05)  [-0.28, -0.10], 0.001 | -0.11 (0.05)  [-0.20, -0.02],  0.013 |
| Non-numeric consequences | -0.12 (0.04)  [-0.19, -0.05], 0.001 | -0.14 (0.04)  [-0.22, -0.06], 0.001 | 0.08 (0.04) [0.00, 0.15], 0.042 | -0.55 (0.05)  [-0.64, -0.46], 0.001 | -0.16 (0.05)  [-0.26, -0.07], 0.001 | -0.19 (0.05)  [-0.29, -0.09], 0.001 | -0.21 (0.05)  [-0.31, -0.11], 0.001 | -0.24 (0.05)  [-0.34, -0.15], 0.001 |
| Arabic-number non-consequences | -0.08 (0.03)  [-0.15, -0.02], 0.013 | -0.11 (0.04)  [-0.18, -0.03], 0.006 | 0.06 (0.04)  [-0.01, 0.13], 0.097 | -0.37 (0.04)  [-0.45, -0.29], 0.001 | -0.10 (0.04)  [-0.19, -0.02], 0.019 | -0.09 (0.045)  [-0.18, -0.001, 0.048 | -0.11 (0.05)  [-0.20, -0.02], 0.018 | -0.18 (0.05)  [-0.26, -0.09], 0.001 |
| LIWC Word count | 0.04 (0.03)  [-0.02, 0.10], 0.157 | 0.004 (0.03)  [-0.06, 0.07], 0.911 | 0.03 (0.03)  [-0.03, 0.10], 0.317 | 0.10 (0.043) [0.01, 0.18], 0.027 | 0.06 (0.04)  [-0.03, 0.14], 0.218 | 0.02 (0.05)  [-0.07, 0.11], 0.699 | -0.02 (0.05)  [-0.11, 0.07], 0.705 | -0.003 (0.04)  [-0.08, 0.08],  0.936 |
| LIWC Emotionality | -0.03 (0.04)  [-0.1, 0.04], 0.359 | -0.04 (0.04)  [-0.11, 0.04], 0.343 | 0.03 (0.05)  [-0.06, 0.12], 0.557 | -0.05 (0.06)  [-0.17, 0.06], 0.397 | -0.03 (0.06)  [-0.15, 0.08], 0.573 | 0.01 (0.07)  [-0.12, 0.15], 0.868 | -0.07 (0.06)  [-0.19, 0.05], 0.258 | -0.06 (0.05)  [-0.16, 0.04],  0.228 |
| Objective numeracy | -0.28 (0.07)  [-0.42, -0.13], 0.001 | -0.13 (0.08)  [-0.28, 0.02], 0.095 | -0.18 (0.07)  [-0.32, -0.04],  0.013 | -0.03 (0.05)  [-0.13, 0.07], 0.519 | -0.04 (0.07)  [-0.18, 0.10], 0.591 | -0.05 (0.08)  [-0.2, 0.09], 0.481 | 0.02 (0.07)  [-0.13, 0.16], 0.819 | -0.09 (0.08)  [-0.24, 0.06], 0.259 |
| Number preferences | 0.13 (0.07)  [-0.02, 0.27], 0.084 | 0.15 (0.08) [0.00, 0.30], 0.049 | 0.02 (0.07)  [-0.12, 0.16], 0.807 | 0.14 (0.05)  [0.04, 0.24], 0.006 | 0.26 (0.07) [0.12, 0.40], 0.001 | 0.26 (0.08) [0.12, 0.41], 0.001 | 0.25 (0.07) [0.10, 0.39], 0.001 | 0.33 (0.08)  [0.18, 0.48], 0.001 |
| Ideology | 0.26 (0.07) [0.13, 0.40], 0.001 | 0.32 (0.07) [0.18, 0.47], 0.001 | -0.02 (0.07)  [-0.15, 0.12], 0.812 | 0.18 (0.05) [0.09, 0.27], 0.001 | 0.56 (0.07) [0.43, 0.70], 0.001 | 0.58 (0.07) [0.44, 0.72], 0.001 | 0.46 (0.07) [0.32, 0. 60], 0.001 | 0.45 (0.07)  [0.30, 0.59], 0.001 |
| Verbal-number consequences x Objective numeracy | -0.04 (0.04)  [-0.10, 0.03], 0.305 | -0.04 (0.04)  [-0.12, 0.04], 0.299 | 0.01 (0.04)  [-0.06, 0.08], 0.763 | -0.06 (0.04)  [-0.15, 0.02], 0.141 | -0.04 (0.05)  [-0.13, 0.05], 0.414 | -0.02 (0.05)  [-0.11, 0.07], 0.682 | -0.04 (0.05)  [-0.13, 0.06], 0.431 | -0.03 (0.05)  [-0.12, 0.06], 0.553 |
| Non-numeric consequences x Objective numeracy | -0.04 (0.04)  [-0.11, 0.03], 0.234 | -0.08 (0.04)  [-0.15, 0.00], 0.058 | 0.09 (0.04) [0.02, 0.17], 0.009 | -0.09 (0.04)  [-0.17,-0.00],  0.042 | -0.05 (0.05)  [-0.14, 0.04], 0.235 | -0.02 (0.05)  [-0.11, 0.07], 0.644 | -0.08 (0.05)  [-0.18, 0.01], 0.085 | -0.10 (0.05)  [-0.19, -0.01], 0.032 |
| Arabic-number non-consequences x Objective numeracy | -0.03 (0.03)  [-0.09, 0.04], 0.425 | -0.05 (0.04)  [-0.13, 0.03], 0.207 | 0.08 (0.04) [0.01, 0.15], 0.029 | -0.08 (0.04)  [-0.17, 0.00], 0.04 | 0.01 (0.04)  [-0.08, 0.09], 0.889 | -0.01 (0.05)  [-0.10, 0.08], 0.836 | -0.05 (0.05)  [-0.14, 0.05], 0.338 | -0.04 (0.05)  [-0.13, 0.05], 0.378 |
| Verbal-number consequences x Number preferences | -0.02 (0.04)  [-0.09, 0.05], 0.557 | -0.03 (0.04)  [-0.11, 0.05], 0.462 | 0.01 (0.04)  [-0.07, 0.08], 0.835 | -0.06 (0.04)  [-0.15, 0.03], 0.161 | 0.03 (0.05)  [-0.06, 0.12], 0.568 | 0.01 (0.05)  [-0.08, 0.10], 0.845 | 0.03 (0.05)  [-0.07, 0.13], 0.540 | -0.02 (0.05)  [-0.12, 0.07], 0.607 |
| Non-numeric consequences x Number preferences | -0.03 (0.04)  [-0.10, 0.04], 0.434 | -0.07 (0.04)  [-0.15, 0.007], 0.074 | -0.06 (0.04)  [-0.13, 0.01], 0.086 | -0.09 (0.04)  [-0.18, -0.01], 0.033 | -0.04 (0.05)  [-0.13, 0.05], 0.332 | -0.07 (0.05)  [-0.16, 0.02], 0.135 | -0.03 (0.05)  [-0.12, 0.07], 0.542 | -0.07 (0.05)  [-0.16, 0.02], 0.150 |
| Arabic-number non-consequences x Number preferences | -0.05 (0.03)  [-0.11, 0.02],  0.167 | -0.02 (0.04)  [-0.10, 0.06], 0.599 | -0.04 (0.04)  [-0.11, 0.03], 0.224 | -0.08 (0.04)  [-0.16, 0.003], 0.061 | -0.10 (0.04)  [-0.19, -0.02], 0.020 | -0.10 (0.05)  [-0.19, -0.01], 0.033 | -0.05 (0.05)  [-0.14, 0.05], 0.328 | -0.09 (0.05)  [-0.18, 0.00], 0.041 |
| Verbal-number consequences x Ideology | -0.07 (0.03)  [-0.14, -0.01], 0.030 | -0.06 (0.04)  [-0.13, 0.02], 0.127 | -0.05 (0.04)  [-0.12, 0.01], 0.122 | -0.03 (0.04)  [-0.12, 0.05], 0.416 | -0.04 (0.04)  [-0.13, 0.05], 0.366 | -0.01 (0.05)  [-0.10, 0.08], 0.757 | -0.07 (0.05)  [-0.16, 0.03], 0.156 | -0.01 (0.05)  [-0.10, 0.08], 0.790 |
| Non-numeric consequences x Ideology | -0.11 (0.03)  [-0.17, -0.04], 0.002 | -0.06 (0.04)  [-0.13, 0.02], 0.131 | -0.03 (0.03)  [-0.10, 0.03], 0.336 | -0.11 (0.04)  [-0.19, -0.03], 0.005 | -0.07 (0.04)  [-0.15, 0.02], 0.115 | -0.05 (0.05)  [-0.14, 0.04], 0.238 | -0.10 (0.05)  [-0.18, -0.01], 0.037 | -0.02 (0.04)  [-0.11, 0.06], 0.591 |
| Arabic-number non-consequences x Ideology | -0.04 (0.03)  [-0.10, 0.03], 0.255 | 0.03 (0.04) [-0.05, 0.10], 0.475 | -0.03 (0.04)  [-0.10, 0.04], 0.366 | -0.08 (0.04)  [-0.161, 0.00], 0.052 | -0.002 (0.04) [-0.09, 0.08], 0.967 | -0.02 (0.05)  [-0.11, 0.06], 0.590 | -0.04 (0.05)  [-0.13, 0.05], 0.388 | -0.01 (0.05)  [-0.09, 0.08], 0.897 |
|  |  |  |  |  |  |  |  |  |
| Nakagawa Rm (fixed effects) | 0.281 | 0.256 | 0.131 | 0.264 | 0.394 | 0.386 | 0.321 | 0.344 |
| Nakagawa Rc (entire model) | 0.811 | 0.784 | 0.787 | 0.598 | 0.756 | 0.756 | 0.719 | 0.751 |

# **Fig. S1. Moderation Analyses of Perceptions of Tweet Coming from an Expert by Objective Numeracy, Number Preferences, and Ideology for Study 2.**

Estimated means and standard errors (+- 1 SE) for the Tweet coming from an expert for each condition by (a) objective numeracy, (b) number preferences, and (c) ideology. Values for continuous variables not on the x-axis, were set to their mean values.

# **Fig. S2. Moderation Analysis of Sharing by Ideology for Study 2.**

Estimated means and standard errors (+- 1 SE) for sharing Tweets by ideology for each condition. Values for continuous variables not on the x-axis, were set to their mean value.

# **_____________________________________________________________________________________________________________________________________________________________________________________________________________________________________________________________________________________________________________________________________________________________________________________________________________________________________________________________________________________________________________________________________________________________________________________________________________________________________________________________________**

# **Supplemental Text 3. Preregistered Analyses for Study 2: Within-Participant Tweet Experiment**

In pre-registered analyses controlling for objective numeracy, participants engaged more with Arabic-number-consequence Tweets. They were more likely to share Tweets containing Arabic-number-consequence information than any other Tweet type; they also wanted to find out more about them (see Tables S2, S3, S4). For example, 21.1% of participants were at least “somewhat likely” to share Arabic-number consequences Tweets compared to participants who saw verbal-number consequences, non-numeric consequences, and Arabic-number non-consequences Tweets (respectively, 18.2%, 15.6%, and 17.6%).

Consequence-number Tweets also were perceived as more trustworthy and likely to be from an expert than other Tweets. Furthermore, in exploratory analyses, they were generally perceived as more accurate, clear, and interesting. Overall, more numerate participants were less likely to share Tweets (b = -0.262, p<.001); no other significant numeracy effects emerged.

# **Table S16. Full Preregistered Model Results for Study 2**

b-coefficients (SE) [CI], t(df), p-values) of preregistered hypotheses concerning fixed effects in mixed model effects (Study 2). Each Tweet type was compared to the Arabic-number consequences Tweets. p<0.001 is indicated as 0.001.

|  | Likely to share | Want to find out more | Came from expert | Trustworthy | Accurate | Clear | Interesting |
| --- | --- | --- | --- | --- | --- | --- | --- |
| Intercept | 2.281 (0.079) [2.126, 2.435], 28.973(202.17), 0.001 | 2.968 (0.085) [2.802, 3.134], 35.054(202.42), 0.001 | 3.171 (0.085) [3.002, 3.340], 37.166(37.78),  0.001 | 4.178 (0.102) [3.978, 4.379], 40.973(94.77), 0.001 | 4.146 (0.107) [3.935, 4.357], 38.635(91.05), 0.001 | 4.547 (0.101) [4.348, 4.745], 45.045(83.95), 0.001 | 4.245 (0.095) [4.059, 4.432], 44.742(156.04), 0.001 |
| Verbal-number consequences | -0.105 (0.033)  [-0.170, -0.040],  -3.175(4019.02), 0.002 | -0.103 (0.038)  [-0.177, -0.029],  -2.740(4020.78), 0.006 | -0.473 (0.041)  [-0.554, -0.393],  -11.508(4052.24), 0.001 | -0.202 (0.043)  [-0.286, -0.118],  -4.714(4023.85), 0.001 | -0.194 (0.045)  [-0.282, -0.107],  -4.34 (4023.90), 0.001 | -0.187 (0.046)  [-0.276, -0.097],  -4.084(4028.26), 0.001 | -0.108 (0.044)  [-0.195, -0.021],  -2.44(4024.04), 0.015 |
| Non-numeric consequences | -0.145 (0.033)  [-0.210, -0.080],  -4.378(4019.93), 0.001 | -0.150 (0.038)  [-0.224, -0.076],  -3.956(4021.81), 0.001 | -0.609 (0.041)  [-0.690, -0.528],  -14.760(4055.50), 0.001 | -0.197 (0.043)  [-0.281, -0.113],  -4.582(4025.12), 0.001 | -0.200 (0.045)  [-0.288, -0.112],  -4.447(4025.17), 0.001 | -0.216 (0.046)  [-0.306, -0.126],  -4.715(4029.83), 0.001 | -0.250 (0.044)  [-0.337, -0.163],  -5.628(4025.31), 0.001 |
| Arabic-number non-consequences | -0.094 (0.033)  [-0.158, -0.029],  -2.825(4018.73), 0.005 | -0.113 (0.038)  [-0.187, -0.039],  -2.987(4020.46), 0.003 | -0.390 (0.041)  [-0.470, -0.309],  -9.465(4051.10), 0.001 | -0.115 (0.043)  [-0.200, -0.031],  -2.687(4023.47), 0.007 | -0.093 (0.045)  [-0.181, -0.005],  -2.080(4023.52), 0.038 | -0.113 (0.046)  [-0.203, -0.024],  -2.477(4027.78), 0.013 | -0.180 (0.044)  [-0.266, -0.093],  -4.048(4023.65), 0.001 |
| Objective Numeracy | -0.262 (0.068)  [-0.395, -0.128],  -3.845(210.00), 0.001 | -0.121 (0.073)  [-0.263, 0.022],  -1.658(210.00), 0.099 | -0.063 (0.041)  [-0.144, 0.018],  -1.525(209.95), 0.129 | 0.032 (0.075)  [-0.115, 0.178], 0.422(209.99), 0.673 | 0.025 (0.078)  [-0.128, 0.177], 0.314(209.99), 0.754 | 0.061 (0.071)  [-0.079, 0.200], 0.857(209.99), 0.393 | -0.029 (0.077)  [-0.18, 0.122],  -0.378(209.99), 0.705 |
|  |  |  |  |  |  |  |  |
| Nakagawa Rm (fixed effects) | 0.211 | 0.098 | 0.204 | 0.06 | 0.056 | 0.07 | 0.064 |
| Nakagawa Rc (entire model) | 0.809 | 0.782 | 0.597 | 0.755 | 0.755 | 0.717 | 0.749 |

# **Table S17. Estimated marginal means (SE) in preregistered analyses for Study 2**

Estimated marginal means (SE) by Tweet type for all primary and secondary dependent measures with objective numeracy set to its mean (pre-registered hypotheses; N = 212). Columns with different letters differed significantly based on Tukey’s adjusted p value < .05. See Table S17 for all comparisons.

|  | Arabic-number consequences  (Tweet *n* = 1069) | Verbal-number consequences  (Tweet *n* = 1061) | Non-numeric consequences  (Tweet *n* = 1058) | Arabic-number non-consequences  (Tweet *n* = 1052) |
| --- | --- | --- | --- | --- |
| **Engagement variables** | | | | |
| Likely to share  (1-5) | 2.281 (0.079)^a^ | 2.176 (0.079)^b^ | 2.136 (0.079)^b^ | 2.187 (0.079)^b^ |
| Want to find out more (1-5) | 2.968 (0.085)^a^ | 2.865 (0.085)^b^ | 2.818 (0.085)^b^ | 2.855 (0.085)^b^ |
| **Opinions about Tweet** | | | | |
| Came from expert (1-5) | 3.171 (0.085)^a^ | 2.698 (0.085)^b^ | 2.562 (0.085)^c^ | 2.781 (0.085)^b^ |
| Trustworthy (1-6) | 4.178 (0.102)^a^ | 3.976 (0.102)^b^ | 3.981 (0.102)^b^ | 4.063 (0.102)^b^ |
| Exploratory analyses of Tweet opinions | | | | |
| Accurate (1-6) | 4.146 (0.107)^a^ | 3.952 (0.107)^b^ | 3.947 (0.107)^b^ | 4.053 (0.107)^ab^ |
| Clear (1-6) | 4.547 (0.101)^a^ | 4.360 (0.101)^b^ | 4.331 (0.101)^b^ | 4.434 (0.101)^b^ |
| Interesting (1-6) | 4.245 (0.095)^a^ | 4.138 (0.095)^a^ | 3.996 (0.095)^b^ | 4.066 (0.095)^a,b^ |

# **Table S18. Full Estimated Means from Preregistered Model Results for Study 2.**

For each primary, secondary, and exploratory variable, estimated marginal means (at the mean level of objective numeracy), standard errors (SE), df, and confidence intervals in each Tweet condition and all contrasts between them including mean difference, SE, df, t-values, Tukey-adjusted p-values, confidence intervals, and effect size indicator (Cohen’s D).

| **Engagement variables** | |  |  |  |  |  |  |  |  |
| --- | --- | --- | --- | --- | --- | --- | --- | --- | --- |
| Likely to share (1-5) | Conditions | Mean | SE | df | lower.CL | upper.CL |  |  |  |
|  | Arabic-number consequences (1) | 2.281 | 0.079 | 202.170 | 2.126 | 2.436 |  |  |  |
|  | Verbal-number consequences (2) | 2.176 | 0.079 | 202.410 | 2.020 | 2.331 |  |  |  |
|  | Non-numeric consequences (3) | 2.136 | 0.079 | 202.650 | 1.980 | 2.291 |  |  |  |
|  | Arabic-number non-consequences (4) | 2.187 | 0.079 | 202.640 | 2.032 | 2.342 |  |  |  |
|  | Contrast | Mean Diff | SE | df | t.ratio | p.value | lower.CL | upper.CL | Cohen's D |
|  | 1-2 | 0.105 | 0.033 | 4019.010 | 3.175 | 0.008 | 0.020 | 0.190 | 0.141 |
|  | 1-3 | 0.145 | 0.033 | 4019.920 | 4.378 | 0.000 | 0.060 | 0.230 | 0.196 |
|  | 1-4 | 0.094 | 0.033 | 4018.720 | 2.825 | 0.025 | 0.008 | 0.179 | 0.126 |
|  | 2-3 | 0.040 | 0.033 | 4020.040 | 1.209 | 0.621 | -0.045 | 0.126 | 0.054 |
|  | 2-4 | -0.011 | 0.033 | 4018.340 | -0.345 | 0.986 | -0.097 | 0.074 | -0.015 |
|  | 3-4 | 0.052 | 0.033 | 4019.320 | 1.552 | 0.407 | -0.034 | 0.137 | 0.069 |
| Want to find out more (1-5) | Conditions | Mean | SE | df | lower.CL | upper.CL |  |  |  |
|  | Arabic-number consequences (1) | 2.968 | 0.085 | 202.410 | 2.801 | 3.135 |  |  |  |
|  | Verbal-number consequences (2) | 2.865 | 0.085 | 202.680 | 2.698 | 3.032 |  |  |  |
|  | Non-numeric consequences (3) | 2.818 | 0.085 | 202.960 | 2.651 | 2.985 |  |  |  |
|  | Arabic-number non-consequences (4) | 2.855 | 0.085 | 202.940 | 2.688 | 3.022 |  |  |  |
|  | Contrast | Mean Diff | SE | df | t.ratio | p.value | lower.CL | upper.CL | Cohen's D |
|  | 1-2 | 0.103 | 0.038 | 4020.780 | 2.740 | 0.031 | 0.006 | 0.200 | 0.122 |
|  | 1-3 | 0.150 | 0.038 | 4021.810 | 3.956 | 0.000 | 0.052 | 0.247 | 0.177 |
|  | 1-4 | 0.113 | 0.038 | 4020.450 | 2.987 | 0.015 | 0.016 | 0.210 | 0.133 |
|  | 2-3 | 0.046 | 0.038 | 4021.940 | 1.221 | 0.613 | -0.051 | 0.144 | 0.055 |
|  | 2-4 | 0.009 | 0.038 | 4020.010 | 0.251 | 0.994 | -0.088 | 0.107 | 0.011 |
|  | 3-4 | 0.037 | 0.038 | 4021.130 | 0.970 | 0.767 | -0.061 | 0.134 | 0.043 |
| **Opinions about Tweet** | |  |  |  |  |  |  |  |  |
| Came from expert (1-5) | Conditions | Mean | SE | df | lower.CL | upper.CL |  |  |  |
|  | Arabic-number consequences (1) | 3.171 | 0.085 | 37.780 | 2.998 | 3.344 |  |  |  |
|  | Verbal-number consequences (2) | 2.698 | 0.085 | 37.840 | 2.525 | 2.871 |  |  |  |
|  | Non-numeric consequences (3) | 2.562 | 0.085 | 37.920 | 2.390 | 2.735 |  |  |  |
|  | Arabic-number non-consequences (4) | 2.781 | 0.085 | 37.890 | 2.609 | 2.954 |  |  |  |
|  | contrast | Mean Diff | SE | df | t.ratio | p.value | lower.CL | upper.CL | Cohen's D |
|  | 1-2 | 0.473 | 0.041 | 4052.260 | 11.507 | <0.001 | 0.368 | 0.579 | 0.511 |
|  | 1-3 | 0.609 | 0.041 | 4055.530 | 14.759 | <0.001 | 0.503 | 0.715 | 0.658 |
|  | 1-4 | 0.390 | 0.041 | 4051.120 | 9.465 | <0.001 | 0.284 | 0.496 | 0.421 |
|  | 2-3 | 0.136 | 0.041 | 4055.980 | 3.278 | 0.006 | 0.029 | 0.242 | 0.146 |
|  | 2-4 | -0.083 | 0.041 | 4049.210 | -2.025 | 0.179 | -0.189 | 0.022 | -0.090 |
|  | 3-4 | 0.219 | 0.041 | 4053.570 | 5.295 | <0.001 | 0.113 | 0.325 | 0.237 |
| Trustworthy (1-6) | Conditions | Mean | SE | df | lower.CL | upper.CL |  |  |  |
|  | Arabic-number consequences (1) | 4.178 | 0.102 | 94.770 | 3.976 | 4.381 |  |  |  |
|  | Verbal-number consequences (2) | 3.976 | 0.102 | 94.880 | 3.774 | 4.179 |  |  |  |
|  | Non-numeric consequences (3) | 3.981 | 0.102 | 95.000 | 3.779 | 4.184 |  |  |  |
|  | Arabic-number non-consequences (4) | 4.063 | 0.102 | 94.990 | 3.860 | 4.266 |  |  |  |
|  | contrast | Mean Diff | SE | df | t.ratio | p.value | lower.CL | upper.CL | Cohen's D |
|  | 1-2 | 0.202 | 0.043 | 4023.850 | 4.714 | <0.001 | 0.092 | 0.312 | 0.210 |
|  | 1-3 | 0.197 | 0.043 | 4025.120 | 4.582 | <0.001 | 0.087 | 0.308 | 0.205 |
|  | 1-4 | 0.115 | 0.043 | 4023.470 | 2.687 | 0.036 | 0.005 | 0.226 | 0.120 |
|  | 2-3 | -0.005 | 0.043 | 4025.160 | -0.120 | 0.999 | -0.116 | 0.106 | -0.005 |
|  | 2-4 | -0.087 | 0.043 | 4022.810 | -2.019 | 0.181 | -0.197 | 0.024 | -0.090 |
|  | 3-4 | 0.082 | 0.043 | 4024.240 | 1.893 | 0.231 | -0.029 | 0.192 | 0.085 |
| **Exploratory analyses of Tweet opinions** | |  |  |  |  |  |  |  |  |
| Accurate (1-6) | Conditions | Mean | SE | df | lower.CL | upper.CL |  |  |  |
|  | Arabic-number consequences (1) | 4.146 | 0.107 | 91.050 | 3.933 | 4.360 |  |  |  |
|  | Verbal-number consequences (2) | 3.952 | 0.107 | 91.160 | 3.739 | 4.165 |  |  |  |
|  | Non-numeric consequences (3) | 3.947 | 0.107 | 91.270 | 3.733 | 4.160 |  |  |  |
|  | Arabic-number non-consequences (4) | 4.053 | 0.107 | 91.260 | 3.840 | 4.266 |  |  |  |
|  | contrast | Mean Diff | SE | df | t.ratio | p.value | lower.CL | upper.CL | Cohen's D |
|  | 1-2 | 0.194 | 0.045 | 4023.910 | 4.342 | 0.000 | 0.079 | 0.310 | 0.193 |
|  | 1-3 | 0.200 | 0.045 | 4025.180 | 4.446 | 0.000 | 0.084 | 0.315 | 0.199 |
|  | 1-4 | 0.093 | 0.045 | 4023.520 | 2.080 | 0.160 | -0.022 | 0.209 | 0.093 |
|  | 2-3 | 0.005 | 0.045 | 4025.220 | 0.116 | 0.999 | -0.110 | 0.121 | 0.005 |
|  | 2-4 | -0.101 | 0.045 | 4022.860 | -2.253 | 0.109 | -0.217 | 0.014 | -0.101 |
|  | 3-4 | 0.106 | 0.045 | 4024.300 | 2.363 | 0.085 | -0.009 | 0.222 | 0.106 |
| Clear (1-6) | Conditions | Mean | SE | df | lower.CL | upper.CL |  |  |  |
|  | Arabic-number consequences (1) | 4.547 | 0.101 | 83.950 | 4.346 | 4.748 |  |  |  |
|  | Verbal-number consequences (2) | 4.360 | 0.101 | 84.060 | 4.159 | 4.561 |  |  |  |
|  | Non-numeric consequences (3) | 4.331 | 0.101 | 84.180 | 4.130 | 4.532 |  |  |  |
|  | Arabic-number non-consequences (4) | 4.434 | 0.101 | 84.170 | 4.233 | 4.634 |  |  |  |
|  | contrast | Mean Diff | SE | df | t.ratio | p.value | lower.CL | upper.CL | Cohen's D |
|  | 1-2 | 0.187 | 0.046 | 4028.260 | 4.084 | 0.000 | 0.069 | 0.304 | 0.182 |
|  | 1-3 | 0.216 | 0.046 | 4029.830 | 4.715 | <0.001 | 0.098 | 0.334 | 0.210 |
|  | 1-4 | 0.113 | 0.046 | 4027.780 | 2.477 | 0.064 | -0.004 | 0.231 | 0.110 |
|  | 2-3 | 0.029 | 0.046 | 4029.870 | 0.640 | 0.919 | -0.089 | 0.147 | 0.029 |
|  | 2-4 | -0.073 | 0.046 | 4026.950 | -1.599 | 0.379 | -0.191 | 0.044 | -0.071 |
|  | 3-4 | 0.103 | 0.046 | 4028.740 | 2.234 | 0.114 | -0.015 | 0.221 | 0.100 |
| Interesting (1-6) | Conditions | Mean | SE | df | lower.CL | upper.CL |  |  |  |
|  | Arabic-number consequences (1) | 4.245 | 0.095 | 156.040 | 4.058 | 4.433 |  |  |  |
|  | Verbal-number consequences (2) | 4.138 | 0.095 | 156.270 | 3.950 | 4.325 |  |  |  |
|  | Non-numeric consequences (3) | 3.996 | 0.095 | 156.500 | 3.808 | 4.183 |  |  |  |
|  | Arabic-number non-consequences (4) | 4.066 | 0.095 | 156.490 | 3.878 | 4.253 |  |  |  |
|  | contrast | Mean Diff | SE | df | t.ratio | p.value | lower.CL | upper.CL | Cohen's D |
|  | 1-2 | 0.108 | 0.044 | 4024.040 | 2.435 | 0.071 | -0.006 | 0.222 | 0.108 |
|  | 1-3 | 0.250 | 0.044 | 4025.310 | 5.628 | <0.001 | 0.136 | 0.364 | 0.251 |
|  | 1-4 | 0.180 | 0.044 | 4023.650 | 4.048 | 0.000 | 0.066 | 0.293 | 0.181 |
|  | 2-3 | 0.142 | 0.044 | 4025.410 | 3.192 | 0.008 | 0.028 | 0.256 | 0.143 |
|  | 2-4 | 0.072 | 0.044 | 4023.050 | 1.615 | 0.370 | -0.042 | 0.186 | 0.072 |
|  | 3-4 | 0.070 | 0.045 | 4024.450 | 1.580 | 0.390 | -0.044 | 0.185 | 0.071 |

# **Supplemental Text 4. Preregistered Analyses of Moderators of Tweet-type Effects for Study 2**

We also tested for moderation by introducing objective numeracy, number preferences, and ideology into the mixed effects regression models with each of their interactions with Tweet type (we replaced subjective numeracy with its subscale number preferences as preregistered for exploration because no significant interactions of subjective numeracy with Tweet type emerged). In each analysis, the simple effects of all Tweet types *vs* the Arabic-number consequences condition remained substantially similar to those presented in Table 1 in the main text and are not discussed in the text.

Overall, people lower in numeracy and number preferences (*vs* higher) and those more conservative (*vs* liberal) were affected less by the Arabic-number consequence Tweets.

*Objective numeracy.* The highly numerate shared all Tweets less than the less numerate; their lower engagement may have been driven by their lower interest and lower perceptions that the Tweets came from experts (Fig S3). The highly numerate generally rated Arabic-number consequence Tweets more positively compared to other Tweet types.

*Number preferences.* Responses on this measure were unrelated to Tweet engagement, relating instead to perceptions of the message source and content. For example, those higher in number preferences perceived all Tweets as more likely from an expert but especially precise-consequence-number Tweets (Fig S3b). Similar results emerged for perceived trust and other opinion questions such as accurate, clear, and interesting.

*Political ideology*. Overall, liberals engaged more than conservatives, wanting to find out more than conservatives. They also were more likely to share (Fig S4) and have positive opinions about the Tweets and especially the precise-consequence-number Tweets. Conservatives were not affected as much as liberals by Tweet type although they did perceive precise-consequence-number Tweets as more likely from an expert (Fig S3c).

# **Fig. S3. Preregistered Moderation Analyses for Study 2.**

Estimated means and standard errors (+- 1 SE) for the Tweet coming from an expert for each condition by (a) objective numeracy, (b) number preferences, and (c) ideology. Pre-registered analyses. Continuous variables not on the x-axis were set to their mean values.

# **Fig. S4. Preregistered Additional Moderation Analyses for Study 2.**

Estimated means and standard errors (+- 1 SE) for sharing Tweets by ideology for each condition. Pre-registered analyses. Continuous variables, other than ideology on the x-axis, were set to their mean values

_________________________________________________________________

**Table S19. Intercorrelations Among All Study 2 Variables**.

Pearson and Spearman correlations are used, respectively, for continuous and binary variables.

|  | 1 | 2 | 3 | 4 | 5 | 6 | 7 | 8 | 9 | 10 | 11 | 12 | 13 | 14 |
| --- | --- | --- | --- | --- | --- | --- | --- | --- | --- | --- | --- | --- | --- | --- |
| 1 Verbal-number consequences | 1.000 |  |  |  |  |  |  |  |  |  |  |  |  |  |
| 2 Non-numeric consequences | -0.333*** | 1.000 |  |  |  |  |  |  |  |  |  |  |  |  |
| 3 Arabic-number non-consequences | -0.332*** | -0.331*** | 1.000 |  |  |  |  |  |  |  |  |  |  |  |
| 4 Likely to share | -0.015 | -0.027 | -0.008 | 1.000 |  |  |  |  |  |  |  |  |  |  |
| 5 Want to find out more | -0.016 | -0.025 | -0.014 | 0.641*** | 1.000 |  |  |  |  |  |  |  |  |  |
| 6 Feelings | -0.033* | 0.049** | 0.026 | 0.180*** | 0.120*** | 1.000 |  |  |  |  |  |  |  |  |
| 7 Came from expert | -0.042** | -0.120*** | -0.018 | 0.373*** | 0.321*** | 0.018 | 1.000 |  |  |  |  |  |  |  |
| 8 Trustworthy | -0.039* | -0.017 | -0.013 | 0.451*** | 0.436*** | 0.109*** | 0.519*** | 1.000 |  |  |  |  |  |  |
| 9 Accurate | -0.036* | -0.019 | -0.007 | 0.449*** | 0.429*** | 0.103*** | 0.490*** | 0.881*** | 1.000 |  |  |  |  |  |
| 10 Clear | -0.030 | -0.025 | -0.012 | 0.354*** | 0.323*** | 0.030 | 0.387*** | 0.721*** | 0.700*** | 1.000 |  |  |  |  |
| 11 Interesting | 0.005 | -0.042** | -0.038* | 0.502*** | 0.605*** | 0.081*** | 0.396*** | 0.665*** | 0.645*** | 0.550*** | 1.000 |  |  |  |
| 12 LIWC Word count | 0.144*** | -0.218*** | -0.009 | 0.039* | 0.026 | 0.031* | 0.179*** | 0.052*** | 0.033* | 0.007 | 0.039* | 1.000 |  |  |
| 13 LIWC Emotionality | -0.046** | 0.059*** | 0.009 | -0.041** | -0.037* | -0.005 | -0.064*** | -0.046** | -0.043** | -0.068*** | -0.036* | -0.222*** | 1.000 |  |
| 14 Objective Numeracy | 0.017 | -0.011 | 0.000 | -0.207*** | -0.089*** | -0.113*** | -0.054*** | 0.022 | 0.016 | 0.042** | -0.019 | 0.006 | 0.000 | 1.000 |

* p<0.05, **p<.01, ***p<.001

# **References**

1. R. D’Andrade, J. Dart, The interpretation of r versus r2 or why percent of variance accounted for is a poor measure of size of effect. *J. Quant. Anthropol.* (1990).

2. D. J. Ozer, Correlation and the coefficient of determination. *Psychol. Bull.* **97**, 307–315 (1985).

3. A. K. Montoya, Moderation analysis in two-instance repeated measures designs: Probing methods and multiple moderator models. *Behav. Res. Methods* **51**, 61–82 (2019).

4. A. K. Montoya, A. F. Hayes, Two-condition within-participant statistical mediation analysis: A path-analytic framework. *Psychol. Methods* **22**, 6–27 (2017).

5. R. M. Baron, D. A. Kenny, The moderator-mediator variable distinction in social psychological research: conceptual, strategic, and statistical considerations. *J. Pers. Soc. Psychol.* **51**, 1173–1182 (1986).

6. A. F. Hayes, Beyond Baron and Kenny: Statistical mediation analysis in the new millennium. *Commun. Monogr.* **76**, 408–420 (2009).

7. E. Peters, P. Slovic, Affective asynchrony and the measurement of the affective attitude component. *Cogni Emot*, **21**, 300-329 (2007).
